# Reshaping the systemic tumor immune environment (STIE) and tumor immune microenvironment (TIME) to enhance immunotherapy efficacy in solid tumors

**DOI:** 10.1186/s13045-022-01307-2

**Published:** 2022-07-07

**Authors:** Liangliang Xu, Chang Zou, Shanshan Zhang, Timothy Shun Man Chu, Yan Zhang, Weiwei Chen, Caining Zhao, Li Yang, Zhiyuan Xu, Shaowei Dong, Hao Yu, Bo Li, Xinyuan Guan, Yuzhu Hou, Feng-Ming Kong

**Affiliations:** 1 Department of Clinical Oncology, The University of Hong Kong-Shenzhen Hospital, Shenzhen, Guangdong 518053 China; 2 Shenzhen People’s Hospital (The Second Clinical Medical College, Jinan University; The First Affiliated Hospital, Southern University of Science and Technology), Shenzhen, Guangdong 518020 China; 3 Shenzhen Public Service Platform on Tumor Precision Medicine and Molecular Diagnosis, Shenzhen, Guangdong 518020 China; 4 Key Laboratory of Medical Electrophysiology of Education Ministry, School of Pharmacy, Southwest Medical University, Luzhou, Sichuan 646100 China; 5 Department of Chemical Biology, School of Life and Marine Sciences, Shenzhen University, Shenzhen, Guangdong 518000 China; 6 Royal Victoria Infirmary, Newcastle upon Tyne Hospitals NHS Foundation Trust, Queen Victoria Road, Newcastle upon Tyne, NE1 4LP UK; 7 Faculty of Medical Sciences, Newcastle University, Newcastle upon Tyne, NE1 7RU UK; 8 Department of Clinical Oncology, LKS Faculty of Medicine, The University of Hong Kong, Hong Kong, China; 9 Advanced Energy Science and Technology Guangdong Laboratory, Huizhou, Guangdong 528200 China; 10 Chinese Academy of Sciences Shenzhen Institutes of Advanced Technology, Shenzhen, Guangdong 518055 China; 11 Guangdong Provincial Key Laboratory of Digestive Cancer Research, Scientific Research Center, The Seventh Affiliated Hospital of Sun Yat-Sen University, Shenzhen, Guangdong 518107 China; 12 Department of Pathogenic Microbiology and Immunology, School of Basic Medical Sciences, Xi’an Jiaotong University, Xi’an, Shaanxi 710061 China

**Keywords:** Tumor immune microenvironment (TIME), Systemic tumor immune environment (STIE), Immunotherapy, Radiotherapy, Single-cell transcriptomics

## Abstract

The development of combination immunotherapy based on the mediation of regulatory mechanisms of the tumor immune microenvironment (TIME) is promising. However, a deep understanding of tumor immunology must involve the systemic tumor immune environment (STIE) which was merely illustrated previously. Here, we aim to review recent advances in single-cell transcriptomics and spatial transcriptomics for the studies of STIE, TIME, and their interactions, which may reveal heterogeneity in immunotherapy responses as well as the dynamic changes essential for the treatment effect. We review the evidence from preclinical and clinical studies related to TIME, STIE, and their significance on overall survival, through different immunomodulatory pathways, such as metabolic and neuro-immunological pathways. We also evaluate the significance of the STIE, TIME, and their interactions as well as changes after local radiotherapy and systemic immunotherapy or combined immunotherapy. We focus our review on the evidence of lung cancer, hepatocellular carcinoma, and nasopharyngeal carcinoma, aiming to reshape STIE and TIME to enhance immunotherapy efficacy.

## Overview of the current status and advanced technology in immunotherapy for solid tumors

Immunotherapy is a type of cancer treatment options that boosts the patient's own immune system to eliminate cancer cells which has changed the landscape in oncologic care and clinical trials in many kinds of solid tumors. There were over 70 FDA-approved immunotherapy drugs up to now at April 2022. Currently, more than 3000 immunotherapy-related clinical trials covering more than 50 types of cancers have been registered around the world (https://clinicaltrials.gov/).

Immunotherapy mainly includes cancer vaccines therapy, oncolytic virus therapy, dendritic cell (DC) therapy, adoptive cell therapy, antibody–drug conjugates (ADCs), and immune checkpoint inhibitors (ICIs). Among them, ICIs by using antibodies to programmed cell death protein 1 (PD-1) and programmed death ligand 1 (PD-L1) are the main and most successful immunotherapy candidates thus far [1, 2]. PD-1 is induced on T cells following TCR signaling activation and leads to impaired T cell function and to immune escape upon ligation to its ligand PD-L1. PD-1 inhibitor immunotherapy blocks the interaction between PD-L1 on the surface of tumor cells or antigen-presenting cells and PD-1 on the surface of CD8^+^ T cells. It reactivates the tumor-killing ability of CD8^+^ T cells, thereby exerting anti-tumor effects. The PD-L1 and PD-L2 are the two ligands for PD-1. The PD-L1 inhibitor only blocks the binding of PD-1/PD-L1, while PD-1 inhibitor blocks the bindings of both PD-1/PD-L1 and PD-1/PD-L2 at the same time [3]. Meanwhile, PD-L1 can also bind to other receptors like B7-1 (CD80) [4]. Inhibition of PD-1/PD-L1 binding reactivates the tumor-killing ability of CD8^+^ T cells, thereby exerts anti-tumor effects. Although some reported that PD-1 inhibitors may have better efficacy than PD-L1 inhibitors and a higher incidence of pneumonitis that PD-L1 inhibitors [5], there is no strong evidence showing the superiority of one over the other. We thus elect PD-1 inhibitor as representative of immunotherapy for the following review.

Currently in the clinic, the use of the biomarker-guided PD-1 inhibitor immunotherapy is mainly guided by the PD-1/PD-L1 expression level, tumor mutation burden (TMB), and tumor microsatellite instability (MSI) [6, 7]. In advanced non-small cell lung cancer (NSCLC), PD-1 inhibitor immunotherapy increased the 5-year survival rate from less than 5% to around 16% [8, 9]. For stage IV NSCLC patients with PD-L1 expression ≥ 50%, the 5-year survival rate reached 31.9% [8, 9]. In advanced hepatocellular carcinoma (HCC) without knowledge of PD-L1 expression levels, the median survival time of patients was less than 1 year, and the median survival time with PD-1 inhibitor immunotherapy alone reached 15.6 months [10, 11]. Among Chinese patients administered with PD-L1 inhibitors combined with VEGF inhibitors, the median survival time was up to 24.0 months [12, 13]. In advanced nasopharyngeal carcinoma (NPC), the progression-free survival (PFS) of PD-1 inhibitor immunotherapy alone was 4.7 months and the 6-month PFS rate was 50% [14], while PD-1 inhibitors combined with chemotherapy achieved an objective effective rate of up to 91%, a disease control rate as high as 100%, and an 86% 6-month PFS [15]. Overall, there are significant successes in clinical practice in immune checkpoint inhibitors in many other solid tumors in addition to the above-mentioned cancers [16–18]. Tumor immunotherapy strategies based on immune checkpoint inhibitors and immune cell therapy have become the frontier of oncology research. However, the overall pan-cancer response rate of PD-1 inhibitor alone was only 20% [19], while combination with radiotherapy increased the response rate to 40% [19]. The underlying reasons for such a low response rate are poorly understood.

In the past, tumor immunology research mainly studied the genomic/transcriptomic profile at the cell population level or detected the expression of a few molecules in tumor tissue. However, these strategies cannot fully describe the functions of various cell types or characterize the changes involved in the immune processes of malignant tumors, largely due to the high heterogeneity, complexity, and plasticity of the tumor immune microenvironment (TIME).

One must be kept in mind that cancer progression and their responses to treatment are also directly influenced by systemic tumor immune environment (STIE), i.e., the global immunity of the host, the macro-environment of the host anti-tumor immunity. STIE, in coordination with TIME, determines the host responses to the immunotherapy. Hiam-Galvez et al. [20] (recently published in Nature Review Cancer) described such systemic cancer immunity focusing on the immune cell traffics in the STIE, connecting the STIE with lymphatic organs, largely based on the data from the mouse models. This review aims to illustrate the orchestrated effect of the TIME and STIE, through the use of the modern technologies such as the single-cell technology and spatial transcriptomics, in the context of patient and tumor as a whole, with an emphasis on the major challenges facing cancer immunotherapy. Additionally, it further assesses the immune modulating effects of cancer local radiotherapy on systemic therapy, to explore strategies to reshape TIME and STIE for improved efficacy of tumor immunotherapy.

## The tumor microenvironment (TME), tumor immune microenvironment (TIME), and systemic tumor immune environment (STIE)

Solid tumor is a highly complex tissue containing highly heterogeneous cancer cells that differ in compositions and evolutionary states derived from different upstream mutations and the tumor microenvironment (TME) formed by immune cells, stromal cells, blood/lymphatic vessels, nerve terminals, and extracellular matrix (ECM) which contains various signal molecules and acts as immune modulating microenvironment that continuously reshapes the local immunity. The composition of TME is a key determinant of tumor–host interaction. Tumor immune microenvironment (TIME), compositing different cell groups of the immune system and their interactions in the TME niche, has been known for its key role on the processes of carcinogenesis, cancer progression, and responses to the treatments [21]. TIME can be divided into infiltrated–excluded (“Cold tumors”) in which CD8^+^ T cells were excluded from the tumor center, and infiltrated–inflamed (“Hot tumors”) in which an infiltrated–inflamed TIME was observed, as demonstrated by the increased expression of PD-L1 in tumors and highly activated CD8^+^ T cells expressing GRZB, IFN-γ, as well as the presence of infiltrated–tertiary lymphoid structures (TLSs) that are like lymph nodes, containing B cells, dendritic cells, and Treg cells [21]. “Hot tumors” have more specific molecular markers on the surface that can be recognized and attacked by T cells that can trigger the anti-tumor immune responses. PD-1/PD-L1 inhibitors increase the interaction between conventional dendritic cells (cDCs) and naive T cells in the draining lymph node and co-stimulated with CD28, facilitating the initiation and rapid expansion of new T cell clones with new antigen specificities [22, 23]. Moreover, PD-1/PD-L1 inhibitors also prompt the reproduction of existing T cell clones in circulation. These expanding peripheral T cells eventually infiltrate the tumor in the TIME and express markers of antigen-specific activation and display functional cytotoxicity [20–23]. In addition, CD40 agonism can be used to accomplish productive de novo immune responses. It can actuate cDC activation in the presence of resistance to checkpoint blockade and trigger these new T cell responses to replace exhausted intratumoral clones [20, 21]. Cancer cells with inclusion of a small proportion of cancer stem cell (CSC) interact with TIME immune cells (e.g., macrophages, MDSCs; DCs; NK cells; T cells; B cells), stromal cells (e.g., fibroblasts and cancer-associated fibroblasts) and the ECM within TME and result in immune activation or immunosuppression effects, thereby affecting the proliferation or metastasis of tumor cells [24].

**Fig. 1:**
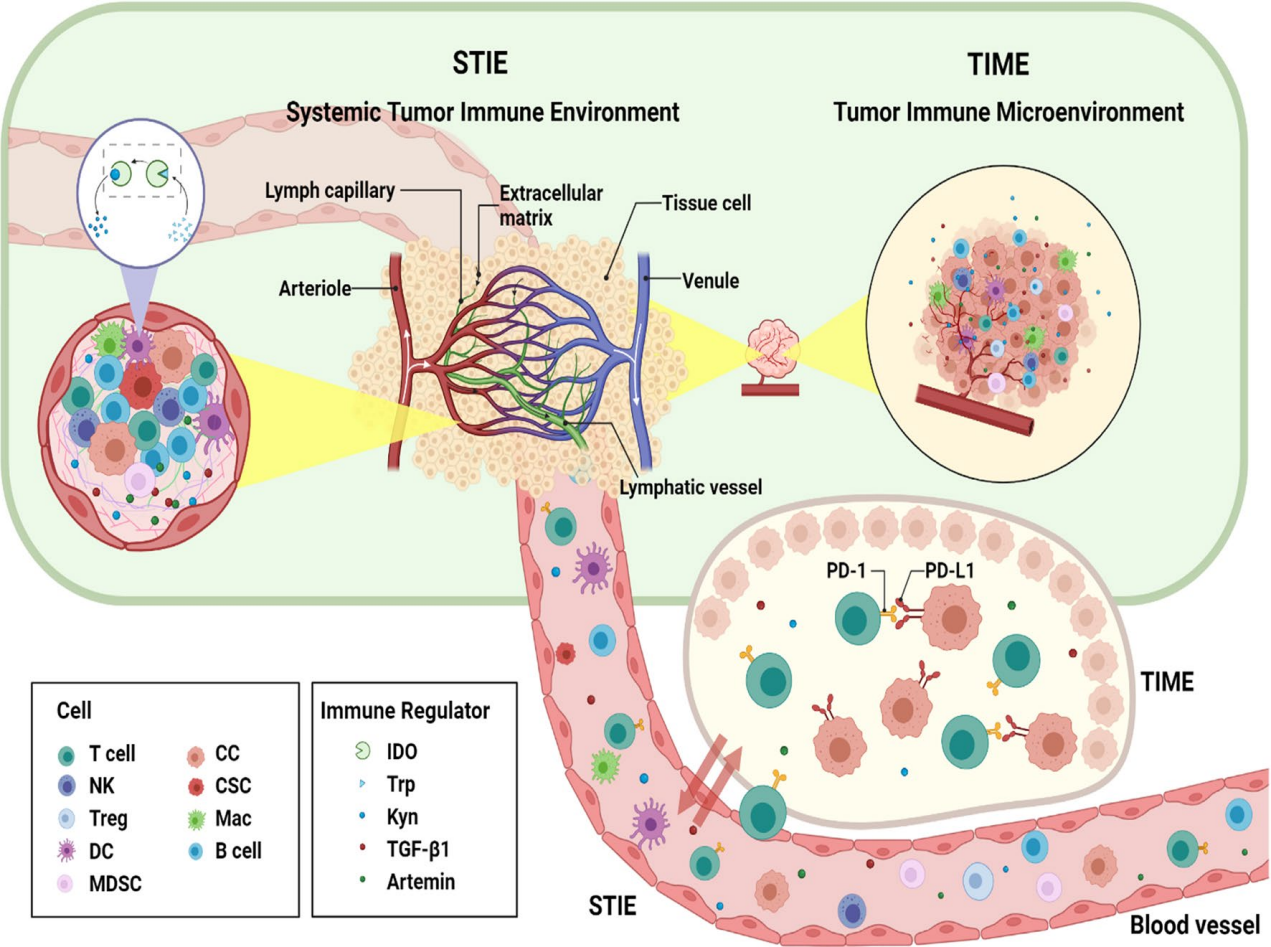
STIE and TIME relationship. The anatomic and interactive relationship between TIME and STIE as well as key components of STIE are shown. STIE circulating in the blood and lymphatic vessels are in close contact with, and directly provide cell and molecular components to the tumor extracellular matrix which can be considered as part of TIME. The major cell and immune regulator components of STIE and TIME may vary with cancer type, examples of non-small cell lung cancer (NSCLC), hepatocellular carcinoma (HCC), and nasopharyngeal carcinoma (NPC) are summarized in Table 1. CC, cancer cell; CSC, cancer stem cell; Mac, Macrophage; DC, Dendritic cell; MDSC, myeloid-derived suppressor cells; NK, natural killer cells; IDO, Indoleamine 2,3-dioxygenase; Kyn, kynurenine; Trp, tryptophan

STIE, primarily controlled by the circulating blood and lymphatic vessels, consisting of the immune modulating molecules and immune cells (Fig. 1), plays an imperative role in communication between the primary tumor site to the distant organs and the host immune organs such as bone marrow and lymph nodes [20]. The functional immune modulators include proteins, cytokines, and metabolites, while the immune cells comprise myeloid cell lineages (neutrophils, monocytes, megakaryocytes, platelets, basophils, eosinophils) and lymphoid cell lineages (T cells, B cells/plasma cells, and NK cells).

There are extensive interactions between TIME and STIE. STIE and TIME are closely related to each other and integrated through capillary vessels and lymphatic drainage system in the tumor. The circulation of lymphatic vessels and blood vessels, which carry immune modulating factors and immune functional cells, is the direct link between the TIME and STIE. Tumors can invade through the blood or lymphatic vessels with the help of immune cells from STIE, and after the infiltration of tumor into the blood or lymphatic vessels, immune cells within the STIE entered the TIME and promoted the metastasis of tumor cells. For example, monocytes derived from the peripheral blood (STIE) could enter the tumor tissue and differentiate into macrophages [25]. Preclinical data from various human and mouse cancer models showed that cancers disrupted the hematopoiesis which subsequently restricted the expansion of immature neutrophils and monocytes in the STIE. Then, this cripple STIE hinders the transport of immune cells to TIME sites which contribute to local immunosuppression [20]. In addition to the commonly studied cellular components of TIME (Fig. 1), immune modulating molecules like TGF-β1, IDO, and Artemin in the ECM have also been considered as important parts of STIE in local tumor, and to play critical roles in cancer development and cancer immunity [26–28]. Many cell components of the TIME are up- or down-regulated (comparison with adjacent normal tissue), while many of the cell components and immune regulators in STIE can be higher or lower than that of the primary tumor, or non-cancer donors. Similarities and variations are also observed from one primary tumor to another, as examples in NSCLC, HCC, and NPC (Table 1). In general, the exhausted T cells increased in the TIME and STIE of tumor patients.

**Table 1:** Major immune components of STIE and TIME

Cancer type	STIE		TIME		REF
	Cell	Immune regulator	Cell	Immune regulator	
NSCLC	↑: CD8^+^ GranB^+^T, Naïve CD4^+^, CD4^+^NKT, Ter cell, NK, Cytolytic CD16^+^NK, CD14^+^ monocyte, basophil -: Neutrophil, CD16^+^ monocyte ↓: CD8^+^PD-1^+^T, CD8^+^T CM, Treg, CD1c^+^DC, Macrophage, Eosinophil, Mast cell	↑: TGF-β1, IDO, Artemin, PD-1, PD-L1, CTLA-4, GITR, IL-17 ↓: BMI1	↑: CD8^+^PD-1^+^ T, CD8^+^ T CM, Treg, Infiltrated CXCR3^+^NK, CD1c^+^DC -: Naïve CD4^+^, Macrophage, CD14^+^monocyte, Neutrophil, Eosinophil ↓: CD8^+^ GranB^+^T, CD16^+^monocyte, NK, CD4^+^NKT, Cytolytic CD16^+^NK, Basophil, Mast cell	↑: TGF-β1, CTLA4, CD11c, CD14, CD39, ICOS, 41BB, IL-6, PPARγ ↓: IL-8, IL-1β, IFN-γ, Granzyme B, CD57, CD86, CD206	[29–40] [29]*
HCC	↑: Treg, MDSC ↓: CD8^+^ T, CD4^+^ T	↑: PD-L1, IL-6, LAYN ↓: PD-1, CTLA4, IFN-γ, LAG3, TIM3, PTPRO	↑: CD8^+^T, CD4^+^T, Treg, B cell, Monocyte, DC, Kupffer cell, Neutrophil ↓: NK, NKT	↑: PD-1, PD-L1, CTLA4, TIM3, LAG3, IFN-γ, IL-10, IgA ↓: CCL4, CCL14	[41–55]
NPC	↑: NK, PD-1^+^ NK -: Treg ↓: CD8^+^T, CD4^+^T, B cell	↑: IL-2, IL-10, IL-18, MMP-9, CR1, IgM	↑: CD8^+^ T, Treg, B cell, PD-1^+^CXCR5^−^CD4^+^ Th-CXCL13, CD19^+^ B cell ↓: NK	↑: PD-1, FOXP3, LAG3, HAVCR2, IFN-γ, IL2RA, IL-18, IL-21, CCL19, CCL20, CXCL10, CXCL13	[56–65]

↑, Upregulation; ↓, Downregulation; -, symbol No significant change in the cited literature studies; ↑, ↓ and - reflected the changes in tumor tissue or cancer patients compared with adjacent normal tissue or non-cancer donors; *: Compared with tumor tissue; *T* T cells, *TCM* central memory T cell, *NK* natural killer, *NKT* natural killer T cell, *DC* dendritic cell, *REF* reference. *NSLC* non-small cell lung cancer, *HCC* hepatocellular carcinoma, and *NPC* nasopharyngeal cancer are shown as examples

The compositions of STIE and TIME are very complex and involve various pathways and mechanisms and vary with the primary tumor types [29–32, 41–51, 56–60] (Fig. 1 and Table 1). Majority of cell and molecular components in the TIME are also present in STIE, at a level similar, higher, or lower relative to TIME. One has to note that the research on cellular molecular level of the STIE is very limited at this time, in particular comparison with the TIME.

The efficacy of immunotherapy relies heavily on the status of STIE and TIME. Immunotherapy augments the anti-tumor immunity in the STIE and TIME and becomes a new strategy for tumor treatment that has brought fundamental changes in the fields of cancer research and cancer care. To summarize the interactive roles of STIE and TIME and clarify their impacts on tumor progression and therapeutic efficacy, we elected to address the following challenges associated with PD-1 inhibitor treatment: (1) an overall low response rate and the heterogeneity of therapeutic efficacy of immunotherapy, (2) the communications between STIE and TIME that establish the environment for cancer metastasis (e.g., brain metastasis), (3) hyperprogressive disease, and (4) treatment-related adverse effects. Additionally, we will take snapshot of the accumulating evidence of the changes of TIME and STIE induced by local radiation therapy (RT) and systemic ICI therapy, to explore the reshaping strategies which may provide strategies to improve the efficacy of immunotherapy by modulating TIME and STIE.

## STIE and TIME on the heterogeneity of immunotherapy efficacy

### STIE on immunotherapy efficacy

The significant impact of STIE on the cancer immunotherapy efficacy was recently comprehensively emphasized by a study from Stanford by Spitzer, demonstrating the key role of STIE on cancer immunotherapy [20]. The investigators performed an organism-wide study in genetically engineered cancer models using mass cytometry and analyzed immune responses in several tissues after immunotherapy and developed intuitive models to visualize single-cell omics data with statistical inference. They reported that: (1) systemic immune activation was evident shortly after effective therapy was administered; (2) during tumor rejection/eradication, only systemic peripheral immune cells sustained their proliferation, (3) an emergent population of peripheral CD4^+^ T cells were significantly expanded in patients responding to immunotherapy. This work emphasized the critical impact of systemic immune responses that drives tumor regression. Recently, a comprehensive review summarized responses of STIE immune cells to ICI treatment [20] demonstrated the critical role of systemic immunity (equivalent to our STIE) for effective natural and therapeutically induced anti-tumor immune responses, with most of the literature from studies of mouse models. In clinic, reduction in circulating lymphocytes, i.e., lymphopenia, an important change in cell component of STIE, has been widely observed and recognized to impact the tumor control and the therapeutic efficacy of various cancers such as lung cancer, breast cancer, pancreatic cancer, melanoma, and sarcoma [66]. In addition, it has been reported that high levels of lymphocytes and low neutrophil-to-lymphocyte ratio (NLR) are associated with better survival of lung cancer patients [67]. In patients with liver cancer receiving immunotherapy, it was noted that those who did not respond to immunotherapy had higher CD4^+^ T cells and Th17 cells and lower CD8^+^ T cells [68]. Viral carcinogenesis-associated HCC and NPC are mainly caused by chronic hepatitis B virus (HBV) and Epstein–Barr virus (EBV), respectively. NPC, an endemic disease usually found in Southeast Asia and North Africa and etiologically linked to Epstein–Barr virus infection, represents a classic 'inflammatory tumor’ that exhibits dense lymphocytes infiltration and high expression of PD-L1 [69]. Stromal cells are in close proximity to and in contact with NPC cells, which could make NPC microenvironment highly heterogeneous and immunosuppressive, preventing tumor cells from being infiltrated by drugs and immune attack, and promote tumor progression. Therefore, the NPC is a heavily immune cell infiltrated cancer with a low degree of differentiation. The high infiltration status of macrophage, plasmacytoid DC (pDC), DC1, NK cell and plasma cells were associated with good prognosis of NPC. There is a dynamic status of T cells from activation to exhaustion in TIME in patients with NPC. The exhausted T cells exhibit features associated with tertiary lymphoid structures (TLS) formation via B cell recruitment and increased abundance of suppressive regulatory T cell (Treg) in the microenvironment, where myeloid recruitment is an NPC-specific event. In addition, T cells entered the TIME and upregulated the activity of IFN-α and IFN-γ response pathways in macrophages, suggesting a potential anti-tumor capacity of these macrophages in NPC [56, 69, 70]. In summary, the composition of TIME explained the mechanism of PD-1 inhibitor immunotherapy efficacy.

### PD-L1 in STIE on immunotherapy efficacy

The circulating immune regulatory factors of the STIE have also been studied as biomarkers in predicting the treatment outcome in clinic. Such effect has been frequently demonstrated in clinical studies. For example, the expression status of PD-1/PD-L1, TGF-β1, and IDO1 was different among individuals even with the same type of cancer. A study of 107 patients with NSCLC revealed that PD-L1 has an observed intratumoral heterogeneity in 78% and inter-tumor heterogeneity in 53% of cases [71]. Plasma soluble PD-L1 (sPD-L1) is another key STIE factor which is elevated in a variety of malignancies and has clinical significance [33]. Okuma et al. [33] reported that majority (75%) of patients with high plasma sPD-L1 levels experienced disease progression in NSCLC treated with the PD-1 inhibitor nivolumab. The time to treatment failure and overall survival (OS) were significantly worse in those with higher plasma sPD-L1 levels when compared to those with lower levels. Similarly, Mazzaschi et al. found that high levels of sPD-L1 negatively affected progression-free survival (PFS) and OS in 109 NSCLC patients treated with ICI [72]. Finkelmeier et al. [73] also found that plasma sPD-L1 levels were positively correlated with liver cirrhosis and cancer stage related in HCC patients who were exposed to PD-1 inhibitor immunotherapy. Indeed, our recent meta-analysis of 8 studies (710 patients) demonstrated that plasma sPD-L1 levels were associated with the clinical outcome of tumor patients and might serve as a predicting biomarker of OS in NSCLC patients given with ICI-based therapies [74].

### TGF-β1 in STIE on TIME and tumor response to PD-1 inhibitor immunotherapy and radiotherapy

**Fig. 2:**
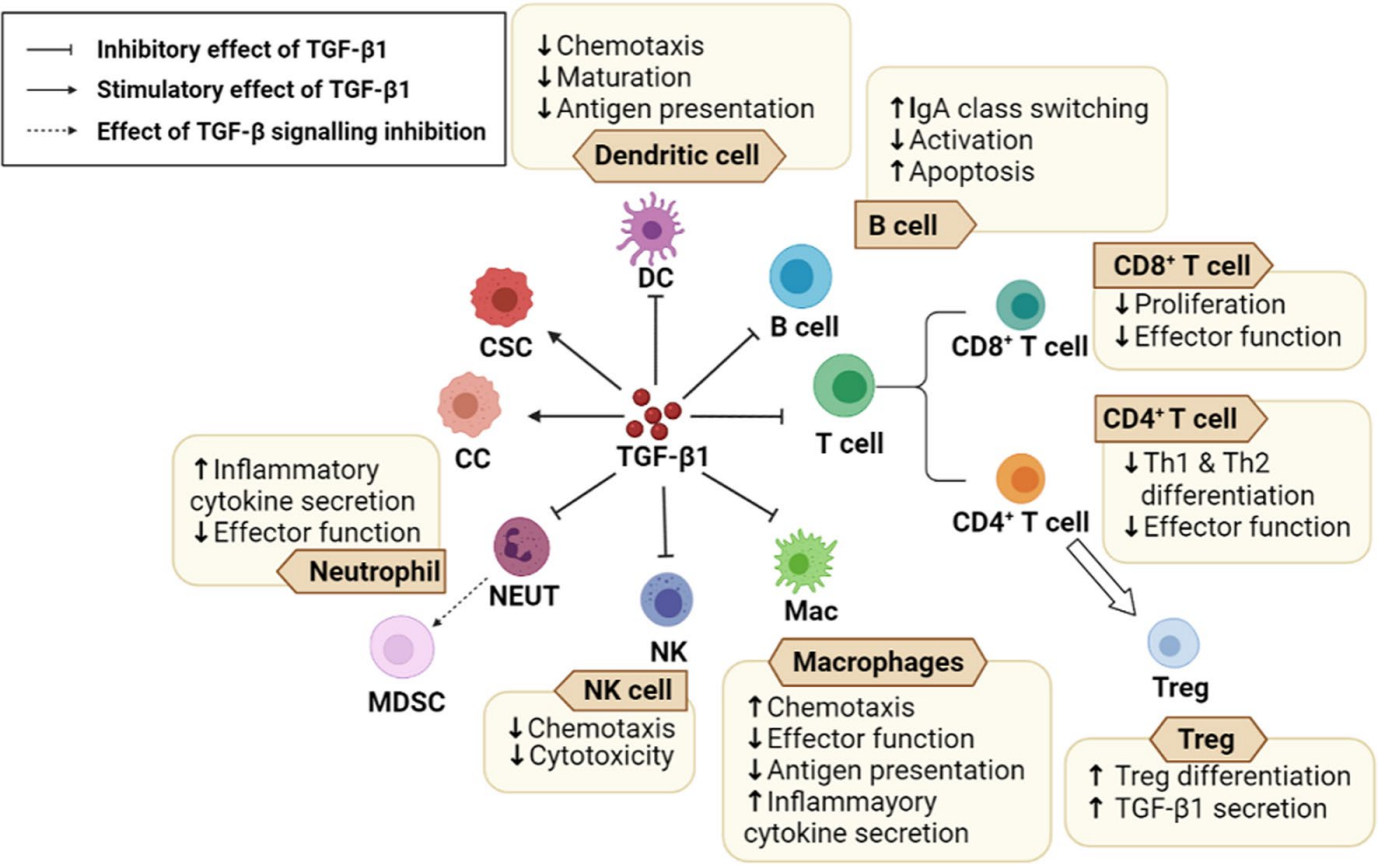
The functions of immune regulator TGF-β1 on TIME and STIE. TGF-β1 has dual roles in the TIME and STIE. TGF-β1 has multiple impacts on different kinds of immune cells. The detail functions of TGF-β1 are shown by different arrows (Solid arrow: stimulation, Dashed arrow: possible stimulation, Vertical-horizontal line: suppression). TGF-β1: Transforming growth factor-beta1; TIME: Tumor Immune Microenvironment; STIE: Systemic Tumor Immune Environment

TGF-β1 is a known immune suppressor which plays a key role in immune modulation. Figure 2 shows the multiple roles of TGF-β1 on TIME and STIE. It inhibits antigen-presenting cells and immune effector cells, like CD8^+^ T cells, and upregulates Treg cells. Most cancer cells have inactivated their epithelial anti-proliferative response and benefit from TGF-β1 by augmenting their gene expression, immunosuppressive cytokine release, and epithelial plasticity [75]. There is also upregulation of TGF-β1 expression and autocrine signaling in cancers [76, 77]. TGF-β1 enhances the invasive ability, stem cell-like characteristics, and therapeutic resistance of cancer cells [78]. In TIME, TGF-β1 produced by cancer cells, stromal fibroblasts, and other cells further promotes cancer progression by shaping tumor structure and inhibiting the activity of anti-tumor immune responses, thereby creating an immunosuppressive microenvironment that prevents or weakens the efficacy of anticancer immunotherapy [78]. As a promoter for carcinogenesis and tumor progression, the function of TGF-β1 varies with the tumor types and the stages of tumor development and the background genetic alterations. In early-stage tumors, TGF-β1 induces apoptosis and restrains the proliferation of cancer cells. Paradoxically, it has tumor-promoting effects in advanced cancer [79]. In addition, TGF-β1 drives tumor stem cell proliferation and contributes to treatment resistance [80]. A study by using mouse model with immune-cold phenotype has demonstrated that the therapeutic combination of TGF-β1 blocking and PD-L1 inhibitor therapy reduced TGF-β1 signal transmission in stromal cells, increased the probability of T cells penetrating into tumor center, and stimulated strong anti-tumor immunity and mediated tumor regression [79].

Our group has previously demonstrated that TGF-β1 in STIE might be a biomarker for tumor progression and survival in lung cancer [34, 81, 82]. Comparing the expression levels of TGF-β1 in plasma of healthy donors and patients with lung cancer, 50% of lung cancer patients had an increased level of TGF-β1 [34], which decreases sharply after radiotherapy [81]. TGF-β1 may be used to predict the long-term outcome of lung cancer patients receiving radiotherapy. Monitoring TGF-β1 level may help predict the persistence and recurrence of the disease after treatment [82]. Meanwhile, we also have demonstrated and validated in patients with NSCLC that the addition of IL-8 and TGF-β1 improved model accuracy for radiation-induced pulmonary toxicity (RILT) [83, 84]. In addition, in 166 inoperable stage I–III NSCLC patients who received chest radiotherapy (≥ 55 Gy) with or without chemotherapy, 14 functionalities of 11 genes in the TGF-β1 pathway were detected via genetic variations of single-nucleotide polymorphisms (SNPs). Four SNPs (SMAD3: rs12102171, BMP2: rs235756, SMAD9: rs7333607, and SMAD4: rs12456284) were found to be significantly associated with the survival of NSCLC patients after radiotherapy [85]. Moreover, the level of circulating TGF-β1 was higher in lung cancer patients and persistent of high level was correlated with poorer prognosis, further suggesting STIE TGF-β1 as an immunosuppressive biomarker [86].

### IDO in STIE on TIME and tumor response to PD-1 inhibitor immunotherapy

**Fig. 3:**
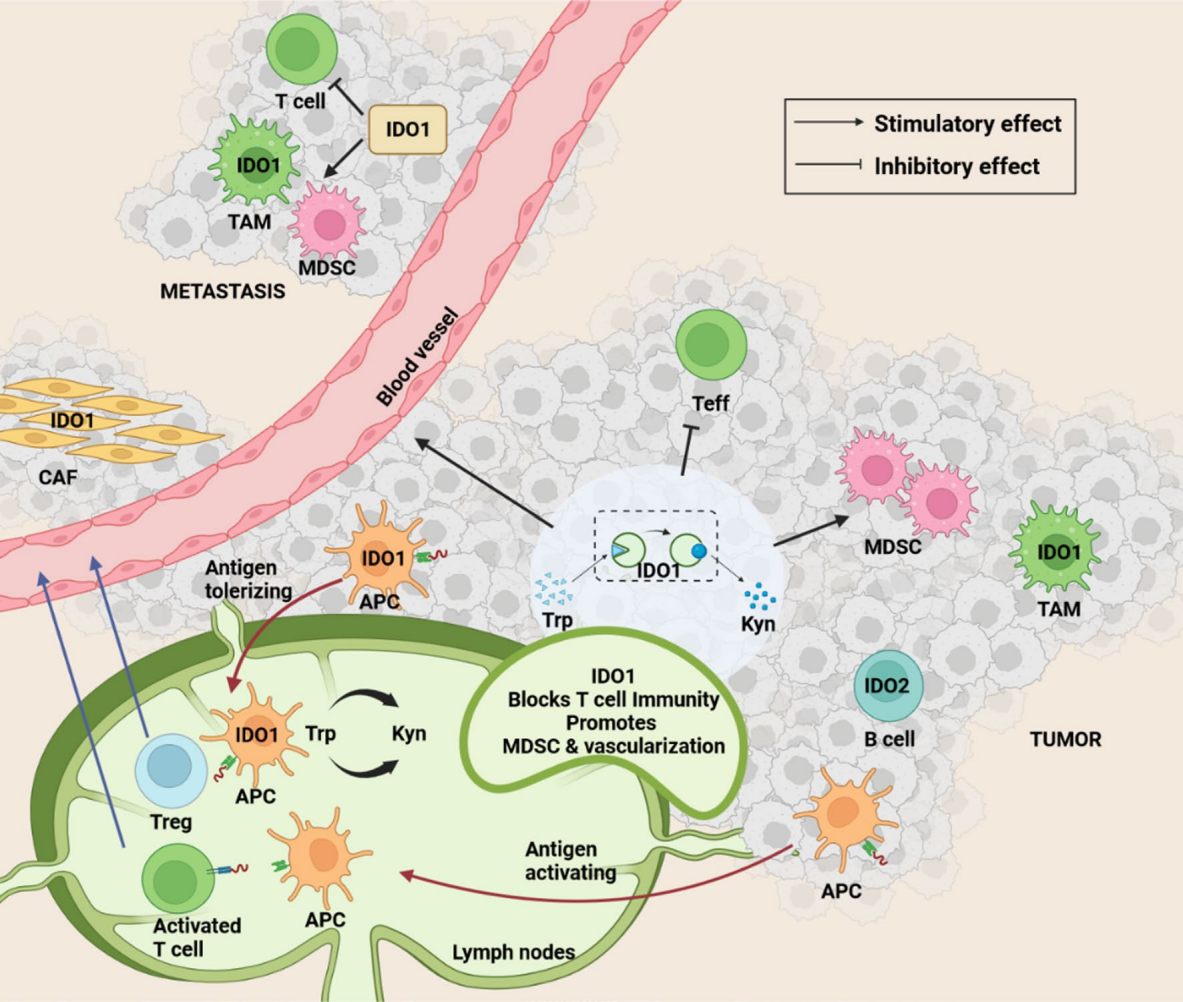
The functions of immune regulator IDO on STIE and TIME. Indoleamine 2,3-dioxygenase (IDO) has multiple roles on STIE and TIME. The detailed functions of IDO in different cells as shown by arrows. IDO1, which suppresses Teff cells and MDSC, mainly catalyzes the breakdown of tryptophan (Trp) to kynurenine (Kyn) in the DC: Dendritic cells, Treg, Activated T cells, and APC cells: Antigen-presenting cells. IDO2 catalyzes in B cells. Solid arrow: stimulation, Dashed arrow: possible stimulation; Vertical-horizontal line: suppression. IDO1 and IDO2 are two different enzymes, that catalyze the same reaction. MDSC: Myeloid-derived suppressor cell; Teff: Effector T cells; CAF: Cancer-associated fibroblast; TAM: Tumor-associated macrophage

IDO, a well-known immunosuppressive immune checkpoint molecule, catalyzes the breakdown of tryptophan (Trp) to kynurenine (Kyn), and its expression is also heterogeneous in STIE [87]. Various IDO inhibitors have been attempted in clinical trials for cancer immunotherapy [88]. It has been shown that IDO1^+^ macrophage population is enriched in patients with NSCLC progression [89]. Figure 3 shows the multiple roles of IDO on TIME and STIE. Markers of IDO activity were reported as predictors of treatment outcomes for NSCLC patients. Botticelli et al. found that higher IDO activity in circulating blood revealed the resistance to PD-1 inhibitor immunotherapy in lung cancer which suggested the potential benefit of PD-1 inhibitor immunotherapy combined with IDO inhibitors in patients with higher IDO [37]. We recently found that among 116 NSCLC patients, changes in IDO metabolites levels after treatment predicted treatment outcomes [90]: (1) Baseline IDO activity was predictive of OS, and higher IDO activity was associated with a higher risk of distant metastasis and shorter survival time; (2) the ratio of kynurenine after/before radiotherapy could be used as biomarker for immune status during radiotherapy. Measurements of these immunomodulatory metabolites could predict OS in patients with NSCLC [90, 91]. In early NSCLC patients receiving definite radiotherapy, elevated IDO activity was associated with poor survival outcomes [90]. According to the IDO levels, the immunosuppressive effects of hypo-fractionated stereotactic body radiotherapy (SBRT) were lower than that of three-dimensional conformal radiation therapy (3D-CRT) [91]. These clinical data are examples that demonstrate the predictive and prognostic potential of STIE immunomodulatory factors in terms of patient and treatment outcomes.

Like TGF-β1, the IDO signaling pathway is upregulated in patients with progressive disease. Bivona et al. [89] compiled a list of 49 clinical biopsy samples from 30 NSCLC patients before and after targeted therapy. Patients with responsive disease showed alveolar regeneration cell signals, suggesting treatment-induced transformations of the original cell state. Patients with disease progression have upregulated IDO signaling pathways. Further studies have found that the failure of combination immunotherapy with IDO inhibitors and PD-1 inhibitors may be due to tumor immune microenvironment status being ignored [89]. A study showed that, under the background of anti-CTLA-4 immunotherapy, host-derived IDO can inhibit the infiltration and accumulation of tumor-reactive T cells in B16 melanoma tumors and weaken the anti-tumor efficacy. Growth of B16 melanoma was considerably delayed compared to wild-type mice, and overall survival rate was improved in IDO knockout mice treated with anti-CTLA-4. IDO deficiency also has synergistic effects with immunotherapy targeting PD-1/PD-L1 and glucocorticoid-induced tumor necrosis factor receptor. Furthermore, the experimental results showed that the overexpressed IDO tumors were resistant to CTLA-4 treatment, but not to CTLA-4/indoximod (IDO inhibitor) combination treatment [92]. There were also reports showing circulating IDO level correlations with overall survival, progression-free survival, and distant metastasis of patients with breast cancer, lung cancer, melanoma, and glioblastoma [90, 91, 93, 94]. These discoveries are informative and may facilitate the design of personalized treatment plans of immunotherapy.

### Artemin in STIE on TIME and tumor response to PD-1 inhibitor immunotherapy

Artemin is a neurotrophic factor in the glial cell line-derived neurotrophic factor (GDNF) family of ligands within the TGF-β superfamily of signaling molecules. Artemin is also involved in the regulation of the tumor progression and tumor responses to cancer therapies, including immunotherapy and radiotherapy. The Artemin receptor is GDNF family receptor alpha-3 (GFRα3) which in itself cannot transmit signals to the cell, but passes signals when binding to Artemin to recruit the co-receptor RET and subsequently enters the cell through RET tyrosine kinase activity [95]. In hepatocellular carcinoma, Artemin inhibits tumor cell apoptosis and promotes its migration by upregulating the expression of TROIBP and ITGB5 [35]. Tumor-inducible, erythroblast-like cells (Ter-cells) with Ter-119^+^CD45^−^CD71^+^ markers deriving from megakaryocyte–erythroid progenitor cells produces Artemin in the spleen and leads high level of Artemin in the blood [35]. Studies have shown that Artemin enhances the resistance of lung cancer cells to radiotherapy through the TWIST1-BCL2 pathway [96], suggesting that Ter cells may inhibit the therapeutic effects of radiotherapy through Artemin. Our group found that radiotherapy significantly reduced the number of tumor-induced Ter cells in the spleen of tumor-bearing mice and the peripheral blood of cancer patients [36]. In addition, in cancer patients and tumor-bearing mice, radiotherapy reduced the level of Artemin in serum and the expression of GFRα3 in tumor tissues [36]. Moreover, our studies also revealed that Artemin significantly inhibited the killing effect of CD8^+^ T cells on tumor cells and reduced the effects of radiotherapy and PD-L1 inhibitor therapy on tumor growth [36]. The impacts of Artemin on PD-L1 inhibitor therapy and the underlying regulatory mechanisms need further research.

### Tumor neoantigens in STIE on TIME and PD-1 inhibitor immunotherapy

STIE also contains tumor neoantigens, which are repertoire of peptides that displays on the tumor cell surface but not normal tissues and serve as strong immune modulators. New technologies that allow a quick screening of neoantigens in each patient and the production of personalized vaccines have broadened the clinical and research prospects. Smith et al. [97] reported 20 patients with resectable non-small cell lung cancer with PD-1 inhibitor before surgical resection. Using single-cell transcriptomics, they found dysfunctional TIL subpopulations that were unable to recognize tumor antigens, including mutation-related neoantigens. Mutation-associated neoantigen (MANA) was using specific T cell assays to identify MANA-specific T cell clones. Tumor neoantigen-specific T cells are related to the effects of PD-1 inhibitor immunotherapy. Therefore, monitoring the changes of neoantigen-specific T cells during PD-1 inhibitor immunotherapy may be helpful to evaluate the response of PD-1 inhibitor immunotherapy. Our group has previously adopted single-cell sequencing to analyze the characteristics of tumor neoantigen-specific T cell subsets and evaluated the correlation between the tumor neoantigen-specific T cells and PD-1 inhibitor immunotherapy response [98]. In our tumor neoantigen study, neoantigens derived from EGFR mutation were identified in patients with lung cancer and found to be related to the survival of the patients [98]. One would assume that MANA or treatment-related changes would play an important role in treatment response of ICIs. Further study is needed on this topic.

### TIME on PD-1 inhibitor immunotherapy efficacy

The heterogeneity of the TIME is clearly one of the most important reasons for the various responses to PD-1 inhibitors. The impact of TIME on PD-1 inhibitor immunotherapy efficacy has been a focus of a recent review [21]. In brief, Chen and Mellman [99] first proposed the concept of “Cancer Immune Cycle,” which simplified tumor immunity into seven end-to-end steps, including (1) the release of tumor antigens, (2) the presentation of antigens, (3) the initiation and activation of T cells, (4) the migration of T cells, (5) the penetration of T cells into tumor tissues, (6) the recognition of tumor cells by T cells, and (7) the elimination of tumor cells. All the above steps are indispensable, function interactively with various components of TIME, and jointly in concert with STIE, outline the complex response of the immune system to tumors. In addition to the genetic differences of the tumor itself, the immune systems of different patients are dissimilar, which is one of the important reasons behind the differences observed in the efficacy of PD-1 inhibitors in different patients [100]. The immune response triggered by effective and ineffective immunotherapy was compared in a triple-negative breast cancer. It was reported that the ineffective group mounted a short-term immune response toward the tumor, while the effective group mounted a coordinated immune response between different tissues including lymph nodes, bone marrow, and blood [101, 102]. Clearly, these phenomena are not explained by the activated immune cells, and one may hypothesize that the immune regulators in the STIE play an important role. The differential responses to immunotherapy heavily rely on each process of the “Cancer Immune Cycle” which also links the interaction between TIME and STIE [103].

### STIE and TIME in brain metastasis and PD-1 inhibitor

**Fig. 4:**
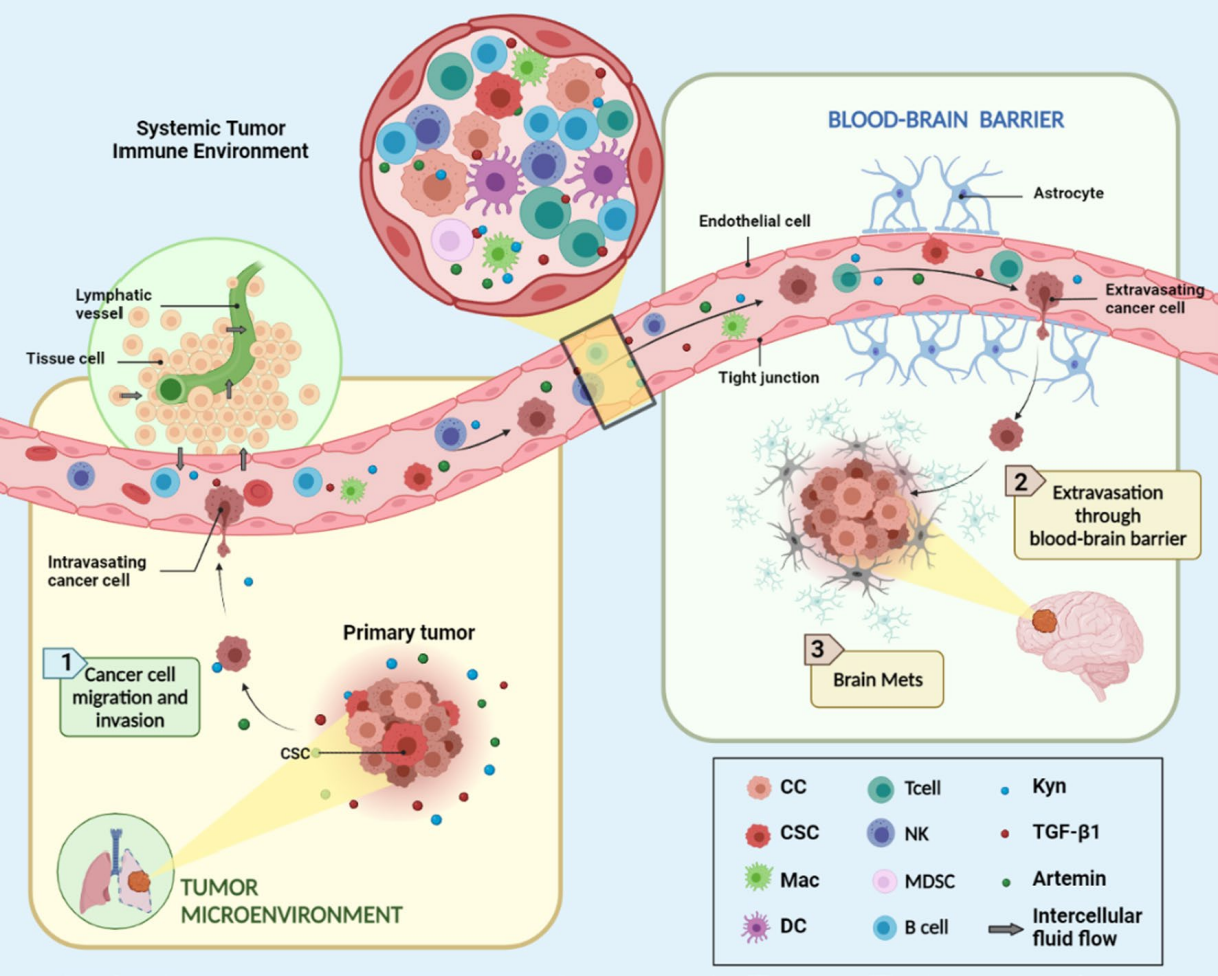
STIE and TIME in brain metastasis. The figure shows the modulating role of systemic tumor immune environment (STIE) on the spread of primary tumor to the brain metastasized sites. It is relevant to local therapy such as radiation therapy (RT) and systemic therapy like PD-1 on the left panel and the components of STIE which include all circulating immune modulating molecules such as cytokines like TGF-β1, IDO biomarkers, Artemin and circulating immune cells such as lymphocytes on the right panel

Cancer metastasis is the major problem associated with cancer mortality. Brain metastasis (BrM) is a major problem for all cancers and accounts for about 20% of patients with solid tumors. Once a patient develops BrM, their prognosis is usually poor with an average survival time of less than 6 months. Nowadays, surgery, chemotherapy, and radiotherapy are the main treatments for BrM patients, though they are mostly not curative. As the toxicities and side effects of the above-mentioned treatment methods are common in brain tissues, PD-1 inhibitor immunotherapy offers a novel approach for treating BrM patients more effectively. The OAK study [104] found that PD-L1 inhibitor monotherapy for NSCLC patients with brain metastases reduced the risk of death compared with chemotherapy (median OS, mOS: 20.1 months vs 11.1 months) and delayed the time of new brain metastases in patients with baseline brain metastases. A meta-analysis of brain metastasis subgroups [105] found that PD-1 inhibitor immunotherapy was beneficial compared with chemotherapy in terms of mOS (8.4 months vs 6.2 months) and the incidence of new intracranial lesions (13% vs 17%) [105–107]. The clinical trials of combination immunotherapy found that PD-1 inhibitor combined with chemotherapy significantly improved the mOS of patients with NSCLC brain metastases when compared with chemotherapy alone (19.2 months vs 7.5 months) [108]. In a study investigating the use of concurrent radiotherapy combined with PD-1 immunization, the inhibitor treatment group had significantly prolonged OS (24.7 months) when compared with the non-synchronous combination therapy group (14.5 months) and the radiotherapy group (12.9 months) (*P* = 0.006) [109]. However, due to the heterogeneity and immunosuppressive characteristics of the TIME of brain metastases and STIE of the patients, the overall effective rate of immunotherapy is still low. The specific mechanism of immunotherapy through the blood–brain barrier is poorly understood. As shown in Fig. 4, the connection between STIE and TIME was established by immune cells and their secreting immune modulating factors such as Kyn, TGF-β1, and Artemin. The STIE and TIME studies for brain metastases are limited. One study showed that the immune cells of brain TME are largely determined by a specific tumor type rather than a central nervous system which has the “immune-privileged niche” [110]. Brain metastases from distant organs contain higher regulator T cells in the TIME. By contrast, gliomas-derived metastases presented abundance of tumor-associated macrophages [110]. A study on brain metastases with PD-1 inhibitor immunotherapy found that the responders had increased T cells and dendritic cells [111]. Indeed, further studies of the mechanisms that regulate the distribution and function of TIME immune cells and STIE in brain metastases are needed to provide a way to improve the immunotherapy efficacy.

### STIE/TIME cancer stem cells and their responses to PD-1 inhibitor

Regression and progression of cancer after PD-1 Inhibitor are clearly a result of a complicated interaction between cancer cells and TIME cells, under a concerted regulation of STIE. Cancer stem cells (CSC), a special group of cells in tumors that maintain self-renewal and can differentiate into other tumor parenchymal cells, can also indirectly promote tumor development by attenuating immune surveillance [112]. CSCs are derived from the non-malignant stem or progenitor cells which are usually inhibited by Notch, WNT, Hedgehog, and Hippo signaling [113–115]. Xenotransplantation experiments have shown that even if one CSC enters the immunodeficient mice, tumors can be formed [116]. In vitro studies have shown that CSC has high expression levels of drug transporters [117, 118] and anti-apoptotic proteins [119, 120], low oxygen free radical levels [121–123], strong ability to repair DNA damage [124, 125], and slow proliferation rate [121, 126]. CSC can stay in a dormant state for a long time with self-renewal ability and plasticity, which can produce heterogeneous tumor cells and contributes to tumor recurrence and metastasis [127, 128]. Studies have shown that CSC expresses high levels of PD-L1 and induces T cell apoptosis by binding to its cognate receptor PD-1 on T cells in breast cancer, colon cancer, head and neck squamous cell carcinoma, and lung adenocarcinoma. More PD-L1 is enriched in CSC abundant patients than non-CSC abundant patients [129]. Currently, targeting Notch, WNT, Hedgehog, and Hippo signaling pathways can inhibit CSC, and a number of clinical trials have been carried out [130]. PD-1 inhibitor immunotherapy combined with targeted CSC therapy improved the effect of anti-tumor therapy [131, 132], which suggests that CSC is one of the potential factors for the failure of PD-1 inhibitor immunotherapy. However, how CSC leads to the failure of PD-1 inhibitor immunotherapy is still unclear. As CSC leads to a highly heterogeneous tumor immune microenvironment, single-cell multi-omics testing is long needed ideal technique to study the role of CSC in TIME. Our unpublished single-cell sequencing data found that CSC could highly express TGF-β1, which recruits MDSC, and subsequently form an immunosuppressive TIME. Meanwhile, PD-L1 was also highly expressed in CSC which aids in immune surveillance evasion, which explains the molecular mechanisms of tumor dormancy. Furthermore, the presence of TGF-β1 and IDO in STIE is heterogeneous, i.e., there are differences in TGF-β1 and IDO in tumors with different malignant degrees, indicating that their responses to treatment (at a proteomic level) were also heterogeneous.

There are many factors and cells in TIME and STIE related to the failure of ICIs. Traditional low-throughput detection methods might not be able to reflect the heterogeneity of the TIME between different tumors and within one tumor. Single-cell transcriptomic and spatial transcriptomic testing of TIME and STIE will provide more insight that may lead to a potential solution. The results of single-cell omics can reveal the response mechanisms of the tumor cells to PD-1 inhibitor immunotherapy and identify predictive biomarkers for more effective treatment.

### STIE and TIME in hyperprogressive disease following PD-1 inhibitor therapy

Hyperprogressive disease (HPD) refers to cases that the volume of the tumor lesion does not decrease but increases after immunotherapy, with a faster disease progression (typically at least two times) compared to before treatment [133, 134]. HPD induced by PD-1 inhibitor immunotherapy is another challenge for immunotherapy alone in addition to their low response rate. Among tumor patients receiving PD-1 inhibitor immunotherapy, 4–29% of patients develop HPD (approximately 13% of lung cancer) [135, 136]. It is unclear that which factors during PD-1 inhibitor immunotherapy contribute to the HPD. One study found that samples from cancer patients experiencing HPD had a greater number of tumor-associated macrophages [137]. It has been suggested that Fc receptors (antibody Fc receptors on the surface of cells such as macrophages) in TIME may be involved in this process. The possible mechanism is checkpoint inhibitor binding to this Fc receptor on macrophages and then inhibits the polarization of macrophages to the M2 subtype, thereby promoting the tumor growth [137].

Cancer genetic variation may play a significant role. Studies have found that 20% of cancer patients with EGFR mutations are more likely to have HPD. This proportion increases to 50% in patients with MDM2 mutations and 67% in patients with MDM4 mutations [138]. Our research team also analyzed the genomic mutation data of tumor patients in the early stage and found that the amplification of CCND1/FGF3/FGF4/FGF19 was prone to develop HPD (unpublished data). In addition, the immune cells and immune regulatory factors in the local tumor environment and circulating blood are directly related to the efficacy of immunotherapy and are also the main contributors to the progression of the disease. We analyzed the STIE of HCC patients treated by immunotherapy and found that patients who did not respond to immunotherapy had higher CD4^+^ T cells and Th17 cells and lower CD8^+^ T cells (unpublished data). If specific molecules involved in HPD are identified, strategies to reduce PD-1 inhibitor immunotherapy-related deaths can be developed to improve the survival rates of potentially responsive patients. Due to concerns about HPD, immunotherapy is frequently restricted to cancer patients without genetic mutations. Moreover, it is important to distinguish the exact roles of PD-1 inhibitors between HPD and pseudoprogression cases.

Single-cell genomics research may identify some novel perspectives of TIME and STIE for this previously unanswered clinical problem. Single-cell transcriptomic data on the patients who undergo HPD show that TGF-β1, IGF-1, ERK/MAPK, and PI3K/AKT signaling pathway were activated in tumors. A subpopulation of innate lymphoid cells named ILC3 cells increased in cancer patients with HPD after PD-1 inhibitor immunotherapy. Besides, the immunogenicity decreased in HPD tumors after therapy [139]. HPD tumors were infiltrated with more PD-1^+^ Treg than PD-1^+^ effector T cells which led to exacerbated growth in gastric cancer [140]. However, there is still a lack of research on the molecular mechanisms behind HPD in PD-1 inhibitor immunotherapy and the biomarkers to predict HPD. The role of STIE and its interaction with TIME deserve further study.

## STIE, TIME, and side effects of PD-1 inhibitor therapy

PD-1 inhibitor immunotherapy can trigger adverse autoimmune reactions, which are called as immune-related adverse events (irAEs), characterized by the infiltration of T cells in various organs, including skin, colon, liver, lung, and endocrine organs. IrAE significantly affects the survival time and quality of life in cancer patients [141]. A pooled analysis of 125 randomized clinical trials involving 20,128 patients found that 2/3 of the patients experienced at least one irAE after PD-1/PD-L1 inhibitor treatment. A small percentage of patients (0.45%) even died of irAE (with pneumonitis being the most common cause of death [28%]) [142, 143].

Cytokines in STIE play a critical role on irAEs, and its significance can be exemplified by the cytokine release syndrome (CRS), a fatal inflammatory reaction after PD-1 inhibitor immunotherapy. Patients with CRS may experience clinical symptoms such as low blood pressure, fever, headache, nausea, and diarrhea, in serious cases with encephalopathy, hypotension, hypoxia, liver dysfunction, and coagulation dysfunction. With the development and widespread use of immunotherapy, more and more tumor patients who received immunotherapy reported to have experience of CRS. In 2017, a patient with extensive metastatic alveolar soft sarcoma who received PD-1 inhibitor immunotherapy developed serious CRS symptoms. This is the first case of cytokine release syndrome in a patient receiving targeted treatment with PD-1 [144]. Gao et al. [145] found that esophageal cancer (ESC) patient developed irAE including CRS after PD-1 inhibitor therapy and had diarrhea, thrombocytopenia, and multi-organ injury. However, in this case, CRS occurred during radiotherapy after PD-1 inhibitor therapy, suggesting that the combination of PD-1 inhibitors and radiotherapy may lead to a greater risk of developing CRS [145]. However, another case report showed that the irAEs of immunotherapy combined with radiotherapy were far less than those of immunotherapy combined with other treatments such as chemotherapy and targeted therapy [146], likely due to various radiotherapy technologies. At present, there are few studies on CRS induced by PD-1 inhibitor immunotherapy under the widespread use of PD-1 inhibitors. It is necessary to carry out relevant evaluations and unravel the underlying immune-related risk factors. The characteristic of STIE and TIME of irAE needs more clear clarification to prevent the irAE.

## Therapeutic strategies to reshape the STIE and TIME

### Reshaping the STIE and TIME with local radiotherapy

**Fig. 5:**
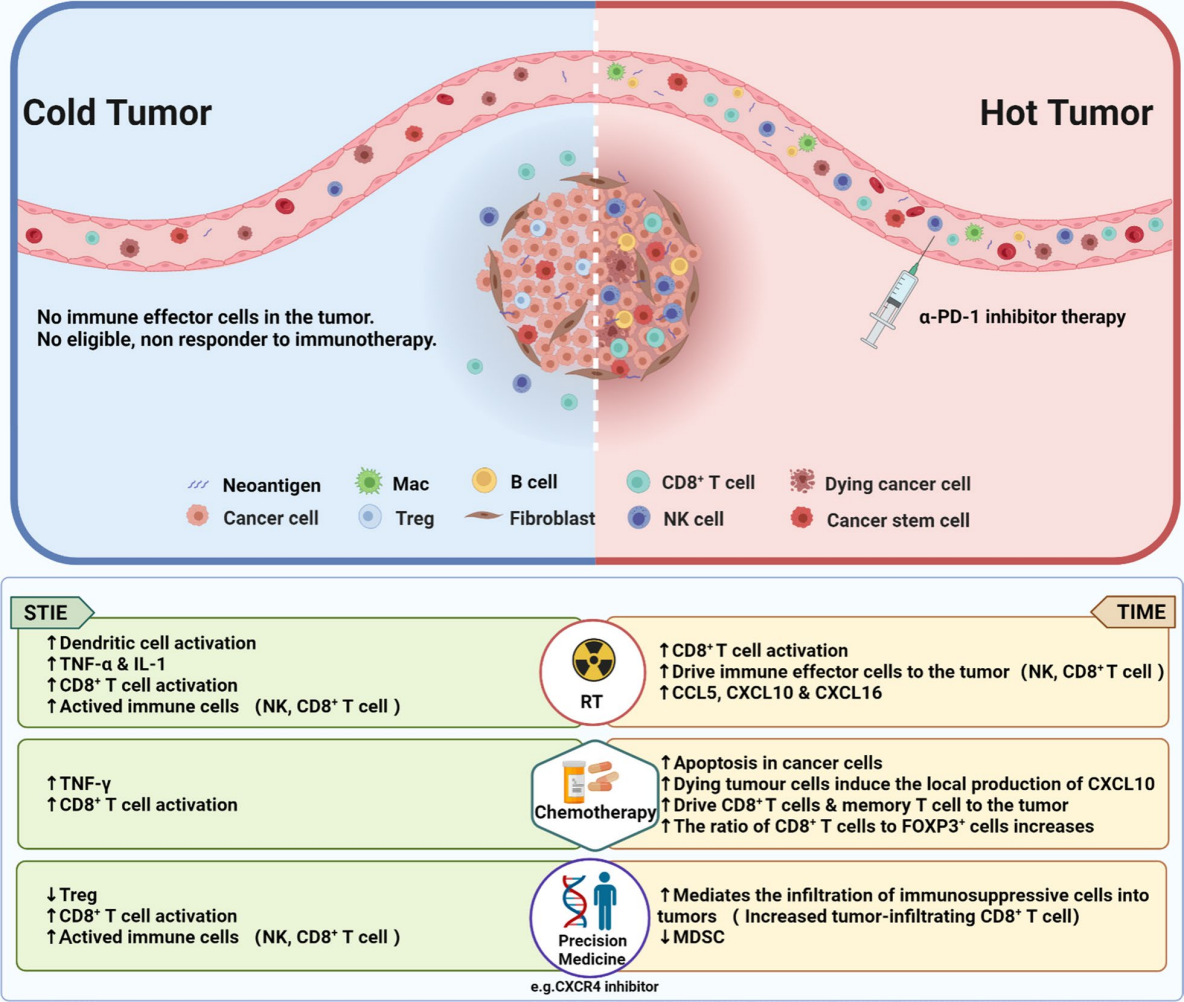
Therapy induced changes in STIE and TIME. The top panel is a simple schema of “cold” and “hot” tumors, and that PD-1 inhibitor is only effective in “hot” tumor. The 3 lower panels illustrate the transformation of the immune environment from the inactive status to the active status through reshaping STIE and TIME by radiotherapy, chemotherapy, and precision medicine therapy (like targeted therapy). In the left part, PD-1 inhibitor immunotherapy is less effective as the local TIME is cold. In the right part, after receiving various types of therapy, the TIME becomes hot. Meanwhile, more activated immune cells such as CD8^+^ T cells appear in the STIE. PD-1 inhibitor immunotherapy can combine with all these therapies to improve the effectiveness of treatment. Multiple immune cells and immune cell-associated factors are involved in this process [153, 154]. STIE: Systemic Tumor Immune Environment; TIME: Tumor Immune Microenvironment

Cancer treatment can induce changes in SITE and TIME which have subsequent effects on immunotherapy. Radiotherapy is one of the strongest treatment modalities to trigger an immune response. Local radiotherapy can modify the TME and TIME and regulate STIE through multiple biologic mechanisms. From the seven end-to-end cancer immune steps of Chen and Mellman [99], radiotherapy can modulate TIME and STIE at least through the following corresponding steps: (1) kill tumor cells to produce immunogenic tumor neoantigens, (2) alter cell surface molecules to facilitate antigen presentation, (3) regulate immune effector cells, (4) induce production of immune modulating cytokines to attract immune potent cells to the tumor sites, (5) help immune cells penetrating into tumor tissues, (6) open the communication between CSC and TIME, and (7) induce production of immune modulating molecules such as TGF-β1 and IDO. Figure 5 demonstrates the transformation of the “Cold” to “Hot” immune environment after radiotherapy. An appropriate dose of focal radiotherapy can stimulate the tumor antigens to CD8^+^ T cells in the proximal TIME and have further effect on the distal sites through STIE [147]. Indeed, radiotherapy can activate anti-tumor immune responses, and combination with radiotherapy has become one of the important ways to improve the effectiveness of PD-1 inhibitor immunotherapy [148]. However, combination therapy with radiotherapy and CTLA-4 inhibitors gave objective responses in only 18% of the enrolled patients and 31% of patients suffered from lung cancer patients [149]. Mechanistically, radiotherapy combined with PD-1 inhibitor immunotherapy can modulate immune function through various biology rationales including activating IFN-β, increasing the activity of CD8^+^ T cells in peripheral blood, and produce an abscopal effect [149]. The specific reshaping functions of radiotherapy on STIE and TIME are shown in Table 2, using NSCLC, HCC, and NPC as examples. In general, radiotherapy disrupted immune cell infiltration and induced de novo inflammation in the TIME. Theoretically, radiotherapy would be beneficial to patients with “Cold” TIME rather than the “Hot” TIME as the radiotherapy may damage existing CD8^+^ T cells [150]. In addition, radiotherapy can prompt the PD-L1 expression in tumor [150] and trigger the activity of STIE through circulating immune cells such as CD8^+^ T cells [53]. Lan et al. [151] also demonstrated that when RT combined with bintrafusp alfa (BA), a bifunctional functional protein targeting PD-L1 and TGF-β1 can reshape the TIME and STIE and stimulate tumor-killing immune reactions. Specifically, BA combined with RT (BART) can enhance the anti-tumor activity, weaken tissue fibrosis, and increase tumor-infiltrating leukocytes in a variety of mouse tumor models with poor immune infiltration. For example, in the KPC mouse model, BART was able to further reduce the 18 F-FDG SUVmax relatives to BA or RT monotherapy and further delay tumor growth. In emT-6 models, BA reduced α-smooth muscle actin (α-SMA), a marker of CAF activation, and collagen deposition, and increased CD8^+^ TIL [152]. Although RT significantly increased α-SMA levels, BA reversed RT-induced α-SMA and collagen deposition. It was also found that BA significantly reduced RT-induced epithelial–mesenchymal transformation (EMT), ECM, fibrosis, and vascular endothelial growth factor (VEGF) expression. Apparently, BA and RT have synergistic activity, that is, BA can further enhance the positive immune modulation effect of RT, while reversing the negative effect of RT to some extent, leading to local remodeling of TME and enhancing the immune response.

**Table 2:** Reshaping STIE and TIME with radiotherapy

Cancer type	STIE		TIME		REF
	Cell	Immune regulator	Cell	Immune regulator	
NSCLC	↑: CD8^+^ T, CD4^+^ T, NK, B cell, CD3^−^ immune cell, γ-H2AX foci PBL -: CD3^+^ T ↓: CTC, NLR, ALC, Ter cell	↑: ssDNA, IFNs, STING/TBK1 pathway, MIP-1α/CCL3 ↓: IDO, Artemin, MDC/CCL22	↑: T cell repertoire, Effector T, DC, N2 neutrophil, M2 macrophage, MDSC -: NK, Treg in TIL ↓: Total lymphocyte, TIL	↑: TGF-β, IFN-γ, PD-L1, ICAM-1, MHC-I, Fas, CSF-1, SDF-1, GFRα3, CCL2, CXCL16, PD-L1 on cancer cell -: PD-1, IFN Receptors, CXCL10, CXCL16	[33, 35, 36, 91, 155–166]
HCC	↑: TNF-α^+^ NK, CD3^+^CD56^+^NKT-like cell ↓: CD4^+^ T	↑: PD-L1, AFP, ALB, TNF-α	↑: CD4^+^ CD25^+^ T, CD4^+^ CD127^+^ T ↓: TIL	↑: TGF-β, MHC-I, PD-L1, PD-L1 on cancer, IFN-γ produced by dLN CD8^+^, CD4^+^ T ↓: HIF-1α	[53, 156, 158, 159, 162, 167–171]
NPC	↑: CCR4^+^ CD8^+^ T	↑: CCL22 ↓: pEBV, miR-142-5p	↓: TIL	↑: TGF-β, PD-L1, MHC-I, PD-L1 on cancer	[156, 158, 159, 172–174]

*CTC* circulating tumor cells, *T* T cells, *NK* natural killer, *DC* dendritic cell, *dLN* tumor draining lymph nodes, *Ter-cells* tumor-inducible, erythroblast-like cells, *NLR* neutrophil-to-lymphocyte ratio, *ALB* albumin, *AFP* alpha-fetoprotein, *ALC* absolute lymphocyte count, *MDC* macrophage-derived chemokine, *PBL* peripheral blood lymphocytes, *TIL* tumor-infiltrating immune cell (e.g., lymphocyte, *APC* antigen-presenting cells), *pEBV* plasma Epstein–Barr virus; ↑, upregulation; ↓, downregulation; -, symbol, no significant change in the cited literature studies; ↑, ↓ and - reflected the changes in tumor tissue or cancer patients compared with adjacent normal tissue or non-cancer donors; *REF* reference

In a joint experiment comparing stereotactic body radiotherapy (SBRT) of hypofractionated treatment with different immune checkpoint inhibitor (CTLA-4, PD-1 inhibitors) therapies, radiotherapy combined with PD-1 inhibitors resulted in longer disease-free survival than that of radiotherapy combined with CTLA-4 inhibitor treatment [149, 155]. In terms of liver metastases, however, Zou et al. [53] found that patients with liver metastases have restricted benefits from immunotherapy with PD-1 inhibitors. In a mouse liver metastasis model, through single-cell transcriptomic analysis for the detection of tumor liver metastasis, activated CD8^+^ T cells in the systemic circulation, the number of peripheral T cells, and the diversity and function of tumor T cells were shown to be decreased [53]. Through the shortage of CD8^+^ T cells and peripheral tolerance mechanisms in the host, liver metastases may cause resistance to adaptive immunotherapy. The combination of liver-directed radiotherapy and PD-1 inhibitor immunotherapy may promote systemic anti-tumor immunity. Liver-directed radiotherapy also reshapes the liver immune microenvironment, prevents the liver siphoning effect of T cells, and restores the therapeutic effect of PD-1 inhibitors in the liver metastasis model.

Our previous work has also demonstrated radiotherapy immunomodulatory effects [67], consistent with results of a meta-analysis of 18 studies (involving 7219 lung cancer patients) [175]. Patients with a high number of lymphocytes and a low ratio of neutrophils to lymphocytes (NLR) in STIE before treatment showed higher overall survival following RT [67]. Analysis of the different stages of tumors found that patients with low NLR have higher survival rates across all stages of tumors [67]. As previously mentioned, the metabolites of IDO changed heterogeneously during radiotherapy: those with IDO activity decreased had significant better survival outcomes and less distant metastasis in lung cancer patients [90, 91]. Radiotherapy modulation effects on IDO immune status were heterogeneously with radiation doses and radiation technology. IDO activity first decreased after low doses of radiotherapy in most patients; at the end of treatment with high dose of treatment, some of them increased and some remained unchanged or decreased; the latter one had better outcome. Patients treated with SBRT (more conformal treatment, less normal tissue radiation therapy) kept IDO level unchanged and had better survival, compared to less conformal 3D-CRT radiotherapy which increased IDO level in more patients [91]. Further studies are also needed to identify the underlying T cell and IDO pathways to answer questions including: which subtypes of T cells are involved in tumor control or progression? What are the factors that control these immune cells? What is the relationship between T cells and IDO metabolites? Studies have found that radiotherapy reduces the total count across all types of lymphocytes, such as CD4^+^ T cells, CD8^+^ T cells, B cells, and NK cells in STIE [176]. The use of STIE and TIME to predict possible adverse immune reactions deserves further study. Our team is currently investigating the changes in TIME and STIE of various types of cancer patients during PD-1 inhibitor immunotherapy and combined PD-1 immunotherapy with radiotherapy.

### Reshaping STIE and TIME with immunotherapy

Immunotherapy can change STIE and TIME. Most of this work is on the fate of T cell which is critical in the process of the reshaping of STIE and TIME with immunotherapy. T cell exhaustion is currently a major obstacle limiting the efficacy of T-cell-based immunotherapy [177]. The developmental hierarchy of exhausted CD8^+^ T cells (Tex) can be divided into four-stage according to transcriptional and epigenetic analyses, namely quiescent resident stage, proliferative circulating stage, circulating mildly cytotoxic stage, and terminally exhausted resident stage. Both quiescent resident and proliferative circulating stages with marked TCF1-TOX were reversible upon immunotherapy [178]. There are three potential reasons to explain the increase in tumor-specific T cell precursors after effective PD-1 inhibitor treatment: 1. reversal of exhausted CD8^+^ T cells, 2. expansion of preexisting precursor cells in the tumor immune microenvironment (TIME), and 3. T cells supplied from outside TIME such as the peripheral blood (STIE) [179]. The reversal of exhausted T cells may not be abundant in the PD-1 treatment responsive group as the previous mouse studies have shown that the epigenetic modifications and characteristics of exhausted T cells are stable and difficult to change [179, 180]. The clonal replacement of T cells was observed in exhausted CD8^+^ T cells and evident in patients with basal or squamous cell carcinoma [181]. In addition, Yost et al. found that the preexisting tumor-specific T cells in TIME were insufficient to reinvigorate and PD-1 treatment-responsive T cells were derived from the distinct clonal T cells which just entered the TIME [181]. Furthermore, Liu et al. found that exhausted T cells were unlikely to be derived from the reinvigoration of terminally exhausted cells; instead, they were accumulated by (1) local expansion and (2) replenishment by peripheral T cells with both new and preexisting clonotypes [179, 181].

Although PD-1 could reinvigorate exhausted CD8^+^ T cells [179, 181, 182], this immune therapy cannot revise the exhaustion-associated epigenetic imprint [180]. Effectors like TOX, TOX2, AP-1, and RGS16 proteins play a key role in the regulation of T cell exhaustion in relation to the transcriptional and epigenetic aspects [177, 183–186]. The specific reshaping of STIE and TIME by immunotherapy is shown in Table 3, using NSCLC, HCC, and NPC as example tumors. The exhausted T cells appeared after PD-1 inhibitor treatment [187–189]. The loss of T cell regulator such as IL-2 regarding as T cell growth factor is accompanied with T cell exhausted phenotype after PD-1 inhibitor treatment in NSCLC [190, 191]. In clinical practice, T cell exhaustion is a major limiting factor of PD-1 and chimeric antigen receptor (CAR)-T cell therapeutics. Weber et al. designed a drug switch to regulate CAR signal to temporarily inhibit T cell activity, which helped preventing CAR-T cell being exhaustion and was able to effectively improve CAR-T cell’s anti-tumor activity in mouse models [192]. Furthermore, combining PD-1 inhibitors with CAR-T immunotherapy can help reverse the effects of Tex.

**Table 3:** Reshaping STIE and TIME with immunotherapy

Cancer type	STIE		TIME		REF
	Cell	Immune regulator	Cell	Immune regulator	
NSCLC	↑: PD-1^+^ CD8^+^ T, Ki-67^+^ PD-1^+^ CD8^+^ T, ICOS^+^ CD4^+^ T, Neoantigen-specific T	↑: IL-2R ↓: Exosomal PD-L1 (Patients responding to PD-1 inhibitor therapy)	↑: Antigen-specific CD8^+^ PD-1^−^ T, ICOS^+^ CD4^+^ T, PD-1^+^ Treg, Texp, NK, Activate PD-L1^+^ NK ↓: CD19^+^ B cell, CD8^+^ T	↑: TGF-β, IFN-γ, TNFα, PD-L1, CD38, CXCL13 on Texp, pSmad3 on cancer ↓: IL-2	[179, 193–204]
HCC	↑: CD8^+^ T, CD3^+^CD56^+^ NKT, CXCR3^+^CD8^+^ T_EM_, Treg, APC	↑: PD-1, TNF-α, IFN-γ, CD107a	↑: TOX^+^ T, CD8^+^ PD1^+^ CXCR^+^ T, TNF^+^ T, CD3^+^CD56^+^ NKT, CD39^+^CD8^+^ TIL	↑: IL-2, CCL4	[54, 55, 205–211]
NPC	↓: EBV-specific T	↑: IFN-γ	↑: IFNβ-dependent NK ↓: CCR4^+^ Treg	↑: TRAIL	[212–214]

*T* T cells, *NK* natural killer, *DC* dendritic cell, *TIL* tumor-infiltrating immune cell (e.g., lymphocyte, *APC* antigen-presenting cells); *T*_*EM*_ effector memory T; *Texp* precursor exhausted T, *EBV* Epstein–Barr virus; *TRAIL* tumor necrosis factor-related apoptosis-inducing ligand, ↑, upregulation; ↓, downregulation; -, symbol: no significant change in the cited literature studies; ↑, ↓ and - reflected the changes in tumor tissue or cancer patients compared with adjacent normal tissue or non-cancer donors; *REF* reference

### Reshaping the STIE and TIME with chemotherapy, targeted therapy, or combined therapy

Cancer treatment can reshape the STIE and TIME which can conversely impact the curative effect of subsequent immunotherapy (Fig. 5). Systemic therapeutic approaches such as chemotherapy can also turn “cold tumors” into “hot tumors” [154]. The reshaping after systemic therapy is apparently complicated and varies with tumor types. Using NSCLC, HCC, and NPC as example tumors, the heterogenous changes of STIE and TIME after cancer systemic chemotherapy and targeted therapy are summarized in Table 4. Chemotherapy has extensive cytotoxic effects on highly proliferating cells, especially on the hematopoietic and immune systems. Chemotherapy can also compromise PD-1 inhibitor therapy, from cytotoxicity on the immune cells [150]. Targeted therapy through small molecular inhibitor mainly functions in the rapidly growing tumor cells. Compared with chemotherapy, targeted therapy inhibits tumor growth without adversely affecting the immune system, making it a potential option in combination with PD-1 inhibitor therapy [150]. It is also noted that chemotherapy agent cisplatin and targeted drugs like crizotinib (ALK and ROS1 inhibitor) can also induce similar immunogenic cell death and both increase CD8^+^ T cell infiltration [215]. For combined immunotherapy, a preclinical study on the Lewis lung carcinoma tumor model showed that combination therapy of IDO inhibitor with radiotherapy can reduce the expression of PD-L1 and Treg and promote the maturation of dendritic cells in the TME. This shows that combined therapy can enhance the activity of T cells and promote anti-tumor immunity [216]. In a study of C57BL/6 xenotransplantation mouse model of lung cancer, although radiotherapy alone increased the infiltration of Treg and cytotoxic T cells, the combination of radiotherapy and PD-1 inhibitor immunotherapy can effectively inhibit tumor progression, increase CD8^+^ T cells, and reduce MDSC and iTreg [217]. The experimental results of an in situ tumor-bearing mouse model showed that after PD-1 inhibitor therapy combined with radiotherapy, the ratio of CD4^+^/CD8^+^ T cells, neutrophils, IFN-γ, TNF, and IL-5 in the lung tissue of the mouse model increased, and inflammatory reactions increased [218]. In the mouse HCC model, the survival rate of PD-1 inhibitor immunotherapy combined with radiotherapy was significantly improved, which greatly inhibited the growth of tumor, increased CD8^+^ T cells, and restored their function [219]. Cytokines and chemokines are soluble proteins produced by a variety of cells, which have a wide range of regulating functions and influence cancer immunotherapy. Reactive CCL4 through inhibiting epigenetic regulators such as HDAC8/SIRT7 with combination of PD-L1 inhibitor therapy may enhance tumor killing in HCC [54, 55]. Interferon-α (IFN-α) could promote tumor apoptosis and inhibit tumor cell proliferation. Interleukin-15 is produced primarily by activated bone marrow cells. Compared with PD-1 inhibitor alone, combined application of PD-1 and LAG-3 inhibitors may improve the anti-tumor function of CD8^+^ T cells [220]. The overactivation of TGF-β1 signal resists the PD-L1 inhibitor therapy in the TIME and STIE [221]. Targeting PD-L1 and TGF-β1 may improve the resistance condition [151]. The first human clinical trial of IL-15 showed that NK and CD8^+^ T cells were amplified in STIE (peripheral blood) of patients with advanced melanoma and patients with renal cell carcinoma (RCC), but with severe adverse reactions [222]. ALT-803 is another variant of IL-15, among 21 patients with NSCLC, 29% achieved objective response and showed elevated levels of circulating NK and CD8^+^ T cells through subcutaneous co-administration of PD-1 inhibitor therapy [223]. TNF-α is mainly produced by monocytes, macrophages, and DCs. It acts as a mediator of anti-tumor immune response in tumor immunotherapy. The results of an experiment in a mouse model showed that TNF-α blockade could overcome resistance to PD-1 inhibitor immunotherapy [224]. Overall, blockade of inhibitory receptors, costimulatory receptors, cytokines, immune checkpoints, and combined use of PD-1 inhibitor with more traditional tumor treatments, such as radiotherapy, chemotherapy, and targeted therapy, have all shown good anti-tumor efficacy, likely through reshaping the TIME and STIE [225].

**Table 4:** Reshaping STIE and TIME with chemotherapy, target therapy, or combined therapy

Cancer type	STIE		TIME		REF
	Cell	Immune regulator	Cell	Immune regulator	
NSCLC	↑: CD8^+^ T, Th1 cell -: PD-1^+^CD8^+^ T, PD-1^+^CD4^+^ T ↓: CD3^+^CD8^+^ T, Treg, Th2 cell, Th17 cell, NK	↑: IFN-γ ↓: ctDNA, IL-4, IL-17	↑: T cell, CD8^+^ T, Senescent CD28^−^CD57^+^ T, Highly differentiated CD8^+^CD28^−^ T, DC -: CD8^+^ TIL density in tumors with a high PD-L1 expression level ↓: CD8^+^ and FOXP3^+^ TIL densities	↑: PD-1, PD-L1, IL-2, CD73, CXCL10 ↓: FOXP3, CTLA4, LAG3, TNFRSF18, CD80	[198, 215, 226–228] [216, 217, 229–231]
HCC	↑: CD14^+^ Monocyte, CD56^+^ NK ↓: CD4^+^CD25^+^Foxp^+^ regulatory T	↑: ST6GAL1, Fas/FasL, MIR30A/15B/107/122/125B/200A/320/374B/645	↑: T cell proliferation and tumor infiltration, CD8^+^ T, CSC ↓: Numbers of tumor vessels and pericytes	↑: IL-1β, CXCL5, HIF-1α/2α ↓: NF‐kB, FGFR4, LIF/JAK1/STAT3, PD-L1/METTL3 on cancer	[211, 228, 232–239] [240, 241]
NPC	↓: CD3^+^ T, CD4^+^ T, CD8^+^ T	↑: CK-19, S-LDH	↑: T cell proliferation, tumor infiltration, CD8^+^ T, NK, PD-1^+^ NK	↑: PD-1, PD-L1, NF-κB, IL-2, CEBPA, miR-3188/PD-L1 on cancer	[228, 242–248]

*T* T cells, *CSC* cancer stem cell, *NK* natural killer, *DC* dendritic cell, *AFP* alpha-fetoprotein, *ALC* absolute lymphocyte count, *TIL* tumor-infiltrating immune cell (e.g., lymphocyte, *APC* antigen-presenting cells); *CK-19* cytokeratin-19, *S-LDH* serum lactic dehydrogenase, *ctDNA* circulating tumor DNA; ↑, Upregulation; ↓, Downregulation; -, symbol: No significant change in the cited literature studies; ↑, ↓ and - reflected the changes in tumor tissue or cancer patients compared with adjacent normal tissue or non-cancer donors; *REF* reference

## Single-cell and spatial transcriptomics in TIME and STIE research

The technique of next-generation sequencing (NGS) has enhanced our understanding of the genomic effects on malignant tumor cells, but cannot accurately dissect individual effects of each TIME cellular components, which worked together to promote the carcinogenesis. Single-cell multi-omics may better analyze the relationship between spatial cell distribution characteristics and gene transcription expression in cancer patients TIME, and their dynamic changes during immunotherapy [249]. Single-cell RNA sequencing (scRNA-Seq) can characterize the transcriptome of each individual single cell and reveal the subpopulations of cells within a given specimen (tumor tissue for TIME or blood sample for STIE) [250]. However, in the necessary tissue dissociation step of scRNA-Seq, the separation of individual cells destroys their spatial positioning in original tissues and their close information to each other. Given that the function of proximal and paracrine signals ranges from 0 to 200 μm, this type of spatial information is essential for understanding the cell-to-cell communication between normal tissues and diseased tissues [250]. Spatial transcriptomics measures all gene activities in tissue samples and maps the locations where the activities occur, which can help us better understand the relationships between gene expression and cell positioning within the tumor tissues, providing the knowledge that single-cell genomics missed. Therefore, strategies combining single-cell transcriptome with spatial transcriptome have a potential to define the TIME and their responses to cancer therapy more precisely [251, 252]. More importantly, the combination of these two technologies may even recover the location of new cell groups in the TIME. Further studies of many cancer patients are needed to examine in-depth understanding of the heterogeneity STIE and TIME as well as dynamic changes after treatment, both of which help to promote precision combination immunotherapy to improve treatment outcome [253–256].

In a recent study of scRNA-Seq analysis in 47 tumors from 36 NSCLC patients, the PD-1 inhibitor immunotherapy-responsive group had significantly increased numbers of precursor exhausted CD8^+^ T cells (Texp), featured by the low expression of co-suppressor molecules and high expression of GZMK [257]. Meanwhile, a study of 29 stage IV NSCLC patients found positive association between the early increase in PD-1^+^ CD8^+^ T cells in the blood and clinical response to the PD-1 inhibitor treatment [258]. Using single-cell transcriptomic sequencing of the primary tumor specimens, a study 12 NSCLC patients treated with immunotherapy combined with EGFR-TKI treatment characterized 15 main cell types (including fibroblasts, endothelial cells, tumor cells, macrophages, T cells, B cells, mast cells, neutrophils, dendritic cells, and ciliated cells). There were high proportion of CD8^+^ T cells and low proportion of the M2/M1 macrophages in the good PFS group [259]. In a study of 7 lung cancer patients undergoing neoadjuvant immunotherapy, single-cell sequencing revealed a higher proportion of tumor-infiltrating T cells in general and lower myeloid cells compared to the group without neoadjuvant therapy. These patients had distinct different expression profiles in terms of leukocyte cell adhesion and cell cycle arrest [260], suggesting the importance of monitoring the TIME in immunotherapy. Another small clinical study of lung cancer samples before and after PD-1 inhibitor treatment found that the CD8^+^ tumor-infiltrating lymphocytes related to cytotoxic function (PRF1, GZMB, and GZMH) and activation (CD38) increased in posttreatment patients [261]. The tumor-associated CD4^+^ T cell clones in STIE peripheral blood have higher cytotoxic activity than the CD8^+^ T cell clones, but post-progressive CD8^+^ T cells can be observed in patients with disease progression. Moreover, T cell abundance decreased significantly, and the proportion of PD-1^+^ T cells decreased [262].

Single-cell sequencing analysis also found that the gene expression profiles were different between the PD-1 high and PD-1 low subpopulations of CD8^+^ T cells in NSCLC, and that the transcription factor TOX can effectively predict the overall survival and PD-1 inhibitor efficacy in NSCLC [263]. Single-cell sequencing of 7 liver cancer patients before and after immunotherapy found that paired tumor biopsies between malignant cells and between tumors of different patients have similar genome specificities, and the expression of SPP1 was significantly increased after treatment. Moreover, the proportion of CD4^+^ memory T cells in the treatment response group was higher [264]. The above data confirmed the utility of single-cell sequencing technology in the in-depth exploration of treatment response, disease progression, and biomarkers during immunotherapy for liver cancer and lung cancer.

Spatial transcriptomics (ST) will overcome the loss in the spatial pattern of transcriptomics of the single-cell sequencing technology. A study applied the ST technology in the tumor tissues with 4 LUAD and 8 LUSC from 12 NSCLC patients, tracked the subclone of the specific sate of tumors through spatial pseudo time, and further described the process of epithelial–mesenchymal transition in detail [265]. Besides, the ST technology of 21 primary tumor tissues from 7 HCC patients found that the fibroblasts and endothelial cells can form a barrier-like capsules to prevent immune cell infiltration which may hinder immunotherapy [266]. In NPC, the efficacy of PD-1 inhibitor treatment can be improved by combination therapy, plus systemic therapy in first-line therapy [267] or radiotherapy [268]. However, due to the difficulty of obtaining samples before and after treatment, the evidence on TIME effect remains limited. Changes in specific immune cell subtype, abundance, time, and spatial correlation in immunotherapy are still obscure and need to be further strengthened. With advances in single-cell technology, studies on STIE and STIE modulating factors may enlighten this field of research quickly.

## Conclusions

In summary, PD-1 inhibitor immunotherapy faces bottlenecks such as overall low response rate, hyperprogressive disease, limited understanding in brain metastasis, and serious irAE. Single-cell transcriptomic and spatial transcriptomic techniques can be used to study the heterogeneities and orchestration between STIE and TIME, before and after treatment with PD-1 inhibitors, the impacts of metabolic neural pathways on TIME, their overall influences on the immune microenvironment, and individual effect of traditional cancer treatment like immune modulating radiotherapy. Studying the TIME of tumor in situ and metastatic sites, along with STIE in blood, is opening a big door for the mechanism of tumor progression and overall cancer treatment. Looking for the key factors in STIE and TIME may identify pathway to improve the treatment efficiency of PD-1 inhibitors. As more clinical trials continue, effective combination of radiotherapy and immunotherapy will continue to make great breakthroughs. More trial evidence with STIE correlative studies is needed to determine the sequence and timing of treatment for maximal clinical benefits.

## Questions and future directions

**Fig. 6:**
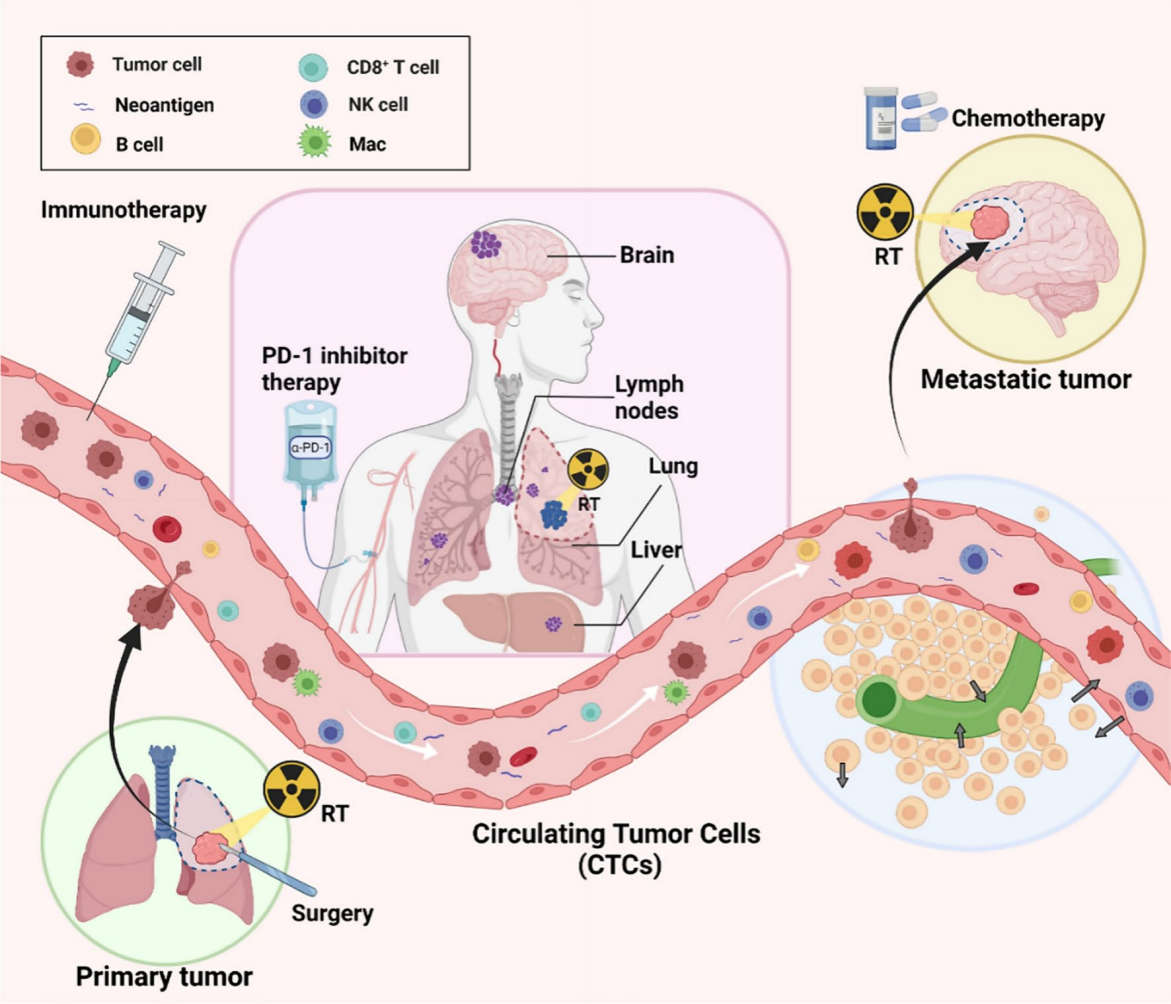
Reshape STIE and TIME to prevent metastasis. This figure shows potential reshaping/targeting points to prevent systemic tumor progression, i.e., metastasis, starting from killing/removing tumors in situ in the primary site through radiotherapy/surgery. Upon the cancer metastasis, systemic therapy such as chemotherapy and immunotherapy are the mainstay treatment for these patients. Radiotherapy is frequently needed for either palliation or consolidation local therapy for good responder and palliation for symptoms for patients with disease progression. Single-cell transcriptomics and spatial transcriptomic techniques are useful to detect these therapeutic effects to uncover the underlying mechanism directly in patients. The red and green pipelines are representing blood vessels and lymphatic vessels which establish the connection between Tumor Immune Microenvironment (TIME) and Systemic Tumor Immune Environment (STIE)

**Fig. 7:**
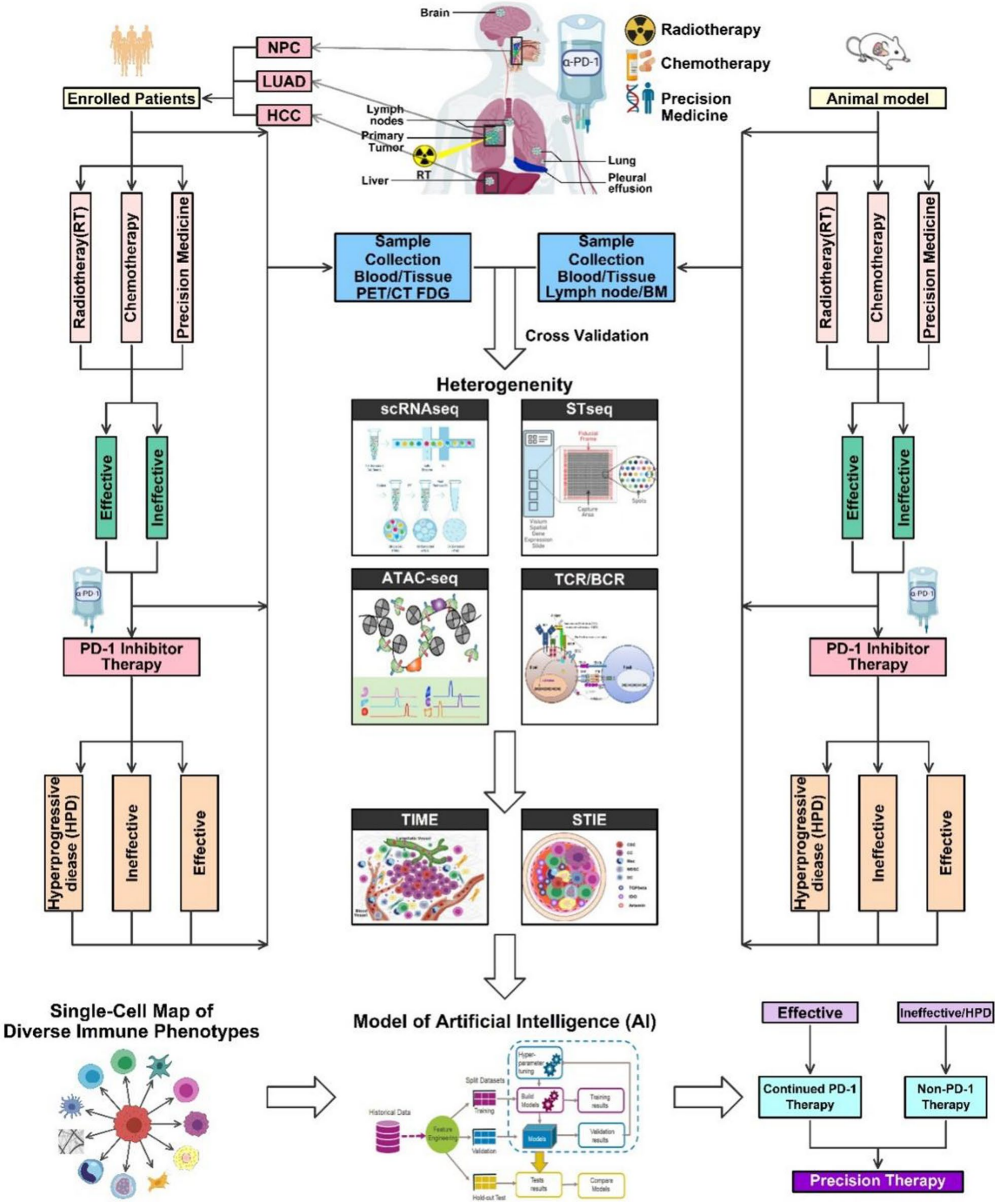
A proposed study schema of STIE and TIME. The pipeline describes a proposed process of studying STIE and TIME including the different treatment strategies and clinical outcomes. The cross-validation of clinical patients and animal models will clearly reveal the STIE and TIME regulation in the RT and PD-1 inhibitor immunotherapy. As the massive data produced by STIE and TIME study, AI model will assist the analysis of data from an in-house or public research

Although new technologies single-cell multi-omics may solve many problems, cancer immunotherapy still faces many challenges. (1) When to use single-cell transcriptomic and spatial transcriptomic techniques to evaluate therapeutic effects of radiotherapy, surgery, chemotherapy, and immunotherapy as Fig. 6 shown? (2) Does PD-1 inhibitor immunotherapy alone or combined with other therapy could reshape STIE and TIME in the same direction? Do TIME and STIE reshape similarly in all cancer patients, and under all various conditions? (3) How to design inhibitors against the key targets in STIE and TIME to improve the treatment efficiency of PD-1 inhibitors? As the STIE and TIME may have opposite directions in anti-tumor therapy as Fig. 1 shown, biomarker-guided precision strategies should be considered. (4) For brain metastases which is associated with large-scope of STIE and TIME plus the blood–brain barrier, how to apply single-cell transcriptomic and spatial transcriptomic techniques to evaluate the key changes of nerve-related genes for improving tumor immunotherapy? In response to these problems, strategies of collecting full spectrum of data in STIE and TIME with inclusion of clinical patient and treatment details as illustrated in Fig. 7 will maximize the learning. As the expensive application of the single-cell transcriptomic and spatial transcriptomic techniques, mounts of integrated data from different centers are commonly used in one study to verify new findings. What’s more, single-cell experiments suffer from “drop out” events due to the randomness of gene expression and the failure of RNA capture or amplification during the sequencing process. It is necessary to use methods such as “Harmony,” “LIGER,” and “Seurat 3” to eliminate batch effects [269]. In addition, mining the informative single-cell transcriptomic and spatial transcriptomic data is a big challenge for researchers; new analysis tools such as artificial intelligence (AI) technology such as deep learning are needed. Nevertheless, we believe that reshaping the STIE and TIME in advanced solid tumors provides a promising approach to enhance efficacy of the immunotherapy. It is encouraging to note that there are a significant number of clinical trials of anti-PD-1/PD-L1 ongoing, with example lists of phase III–IV trials in lung cancer, hepatocellular carcinoma, and nasopharyngeal carcinoma shown in Table 5. With all of these, plus collaborations among multidisciplinary investigators from clinical bedside to the basic benchside, and researchers crossing fields from computer science, biologist, dosimetrist, and physics, we will advance the field to a new horizon of success.

**Table 5:** Ongoing randomized clinical trial of testing combined therapy with PD-1/PD-L1

NCT number	Conditions	Experimental arms	Control arm	Phases	Enrollment	Primary outcome measures
NCT03924869	NSCLC (Stage I/II)	SBRT + Pembrolizumab (Anti-PD-1)	SBRT + Placebo	Phase 3	530	EFS, OS
NCT03774732	NSCLC (Stage IIIB/IIIC/IV)	3D-CRT/SABR + Pembrolizumab (Anti-PD-1) + Chemotherapy	Pembrolizumab (Anti-PD-1) + Chemotherapy	Phase 3	460	OS
NCT05298423	NSCLC (Stage III)	Pembrolizumab (Anti-PD-1)/Vibostolimab Coformulation + Chemotherapy + Thoracic Radiotherapy	Chemotherapy + Thoracic Radiotherapy + Durvalumab (Anti-PD-L1)	Phase 3	784	PFS, OS
NCT03288870	NSCLC (Stage IIIB/TNM Stage 4)	BCD-100 (Anti-PD-1) monotherapy	Docetaxel monotherapy	Phase 2 Phase 3	218	OS
NCT03150875	NSCLC (Advanced/Metastatic)	IBI308 (Anti-PD-1)	Docetaxel	Phase 3	290	OS
NCT03922997	NSCLC (Advanced/Metastatic)	Atezolizumab (Anti-PD-L1)	/	Phase 3	101	SAER
NCT02504372	NSCLC (Stage IB/II-IIIA)	Pembrolizumab (Anti-PD-1)	Placebo	Phase 3	1177	DFS
NCT03285763	NSCLC (Advanced/Metastatic)	Atezolizumab (Anti-PD-L1)	/	Phase 4	619	PAEs
NCT03949231	HCC (Advanced)	PD1/PDL1 inhibitor hepatic artery infusion	PD1/PDL1 inhibitor vein infusion	Phase 3	200	OS
NCT04738487	NSCLC	Pembrolizumab (Anti-PD-1) + Vibostolimab (Anti-PD-1)	Pembrolizumab (Anti-PD-1)	Phase 3	1246	OS, PFS
NCT04331626	NSCLC (Metastatic)	Nivolumab (Anti-PD-1) + Low-dose Gemcitabine	/	Phase 4	50	ORR
NCT04205812	NSCLC (Metastatic)	Retifanlimab (Anti-PD-1) + Chemotherapy	Placebo + Chemotherapy	Phase 3	530	PFS, OS
NCT03594747	NSCLC (Advanced)	Tislelizumab (Anti-PD-1) + Carboplatin + Paclitaxel Tislelizumab (Anti-PD-1) + Carboplatin + Nab-Paclitaxel	Carboplatin + Paclitaxel	Phase 3	360	PFS
NCT03663205	NSCLC (Advanced)	Tislelizumab (Anti-PD-1) + Platinum + Pemetrexed	Cisplatin/Carboplatin + Pemetrexed	Phase 3	334	PFS
NCT04702009	NSCLC (Advanced)	Anti-PD-1/PD-L1 Antibody + Chemotherapy + Bronchoscopy-assisted Interventional Therapy	Anti-PD-1/PD-L1 Monoclonal Antibody + Chemotherapy	Phase 2 Phase 3	80	ORR
NCT03178552	NSCLC (Unresectable/Advanced/Metastatic)	Cohort A: Alectinib 600 Milligrams (mg) Cohort B: Dose Finding Phase (DFP) Alectinib Cohort B: Dose Expansion Phase (DEP) Alectinib Cohort C: Atezolizumab (Anti-PD-1)1200 mg Cohort D: Entrectinib 600 Milligrams (mg) Cohort E: Atezolizumab (Anti-PD-1), Vemurafenib, and Cobimetinib Cohort F: Atezolizumab (Anti-PD-1), Bevacizumab, Carboplatin, and Pemetrexed	Cohort C: Pemetrexed, Cisplatin or Carboplatin Cohort C: Gemcitabine, Cisplatin or Carboplatin	Phase 2 Phase 3	700	PFS, TIR, ORR
NCT03976375	NSCLC (Metastatic)	Pembrolizumab (Anti-PD-1) + Lenvatinib (Anti-VEGF, Anti-FGFR, Anti-PDGFRα) Lenvatinib (Anti-VEGF, Anti-FGFR, Anti-PDGFRα) Monotherapy	Docetaxel	Phase 3	405	OS, PFS
NCT04921358	NSCLC (Advanced/Metastatic)	Tislelizumab (Anti-PD-1) + Sitravatinib (Anti-AXL, Anti-MER, Anti-VEGFR2, Anti-tPDGFR, Anti-KIT, Anti-RET, Anti-MET, Anti-DDR2, Anti-TRKA)	Docetaxel	Phase 3	420	OS, PFS
NCT03906071	NSCLC (Advanced/Metastatic)	Nivolumab (Anti-PD-1) + Sitravatinib (Anti-AXL, Anti-MER, Anti-VEGFR2, Anti-tPDGFR, Anti-KIT, Anti-RET, Anti-MET, Anti-DDR2, Anti-TRKA)	Docetaxel	Phase 3	532	OS
NCT03829332	NSCLC (Metastatic)	Pembrolizumab (Anti-PD-1) + Lenvatinib (Anti-VEGF, Anti-FGFR, Anti-PDGFRα)	Pembrolizumab (Anti-PD-1) + Placebo	Phase 3	623	OS, PFS
NCT04157985	NSCLC HCC (Advanced)	Discontinue Treatment with PD-1/PD-L1-1 inhibitor	Continue Treatment with PD-1/PD-L1 inhibitor	Phase 3	578	PFS
NCT04229355	HCC (Unresectable/Advanced)	DEB-TACE + Sorafenib DEB-TACE + Lenvatinib (Anti-VEGF, Anti-FGFR, Anti-PDGFRα)	DEB-TACE + PD-1 inhibitor	Phase 3	90	PFS
NCT03062358	HCC (Advanced)	Pembrolizumab (Anti-PD-1) + BSC	Placebo + BSC	Phase 3	454	OS
NCT05307926	HCC (Recurrent)	PD-1 inhibitor	TACE	Phase 2 Phase 3	655	DFS, TEAEs
NCT03867084	HCC	Pembrolizumab (Anti-PD-1)	Placebo	Phase 3	950	RFS, OS
NCT04167293	HCC (Early)	SBRT + Sintilimab (Anti-PD-1)	SBRT	Phase 2 Phase 3	116	PFS
NCT04709380	HCC (Advanced)	Radiotherapy + Toripalimab (Anti-PD-1)	Sorafenib	Phase 3	85	TPP
NCT03605706	HCC (Advanced)	SHR-1210(Anti-PD-1) + FOLFOX4	SHR-1210(Anti-PD-1) + Placebo	Phase 3	396	OS
NCT03713593	HCC (Advanced)	Lenvatinib (Anti-VEGF, Anti-FGFR, Anti-PDGFRα) + Pembrolizumab (Anti-PD-1)	Lenvatinib (Anti-VEGF, Anti-FGFR, Anti-PDGFRα) + Placebo	Phase 3	750	PFS, OS
NCT03764293	HCC (Unresectable/Advanced/Metastatic)	SHR-1210 (Anti-PD-1) + Apatinib (Anti-VEGFR2)	Sorafenib	Phase 3	543	OS, PFS
NCT05313282	HCC (Advanced)	Hepatic Arterial Infusion combined with Apatinib (Anti-VEGFR-2) and Camrelizumab (Anti-PD-1)	Apatinib (Anti-VEGFR-2) + Camrelizumab (Anti-PD-1)	Phase 3	140	PFS
NCT03427827	NPC (Advanced)	Camrelizumab (Anti-PD-1)	BSC	Phase 3	442	FFS
NCT04376866	NPC (Recurrent)	CCRT + Toripalimab (Anti-PD-1)	CCRT	Phase 3	204	OS
NCT04778956	NPC (Resectable/Recurrent)	Toripalimab (Anti-PD-1) + Salvage Surgery	Salvage Surgery	Phase 3	218	DFS
NCT04453813	NPC (Recurrent)	Toripalimab (Anti-PD-1) + Concurrent Chemoradiotherapy	Concurrent Chemoradiotherapy	Phase 3	226	PFS
NCT03907826	NPC (Recurrent)	PD-1 antibody + Chemoradiotherapy (IMRT + GP)	Chemoradiotherapy (IMRT + GP)	Phase 3	212	OS
NCT04907370	NPC (Advanced)	Toripalimab (Anti-PD-1) + Induction Chemotherapy + IMRT	Toripalimab (Anti-PD-1) + Induction Chemotherapy + CCRT	Phase 3	520	FFS
NCT04557020	NPC (Advanced)	PD-1 antibody + Chemotherapy + IMRT	Chemotherapy + IMRT	Phase 3	200	PFS
NCT03700476	NPC (Advanced)	Sintilimab (Anti-PD-1) + Chemotherapy + IMRT	Chemotherapy + IMRT	Phase 3	425	FFS
NCT05097209	NPC (Advanced)	Camrelizumab (Anti-PD-1) + Chemotherapy + IMRT	Chemotherapy + IMRT	Phase 3	458	PFS
NCT05340491	NPC (Recurrent)	Chemotherapy + IMRT	Chemotherapy + IMRT	Phase 3	212	OS
NCT04453826	NPC (Stage IVa, Stage II-III)	Camrelizumab (Anti-PD-1) + chemoradiotherapy arm	Chemoradiotherapy alone	Phase 3	388	PFS
NCT04890522	NPC (Metastatic)	Triprilimab (Anti-PD-1) + Cisplatin + 5-Fluorouracil	Triprilimab (Anti-PD-1) + Cisplatin + Gemcitabine	Phase 2 Phase 3	622	OS, PFS
NCT05342792	NPC (T4N + or TanyN2-3M0)	PD-1 antibody + Metronomic Capecitabine (Chemotherapy)	Metronomic Capecitabine (Chemotherapy)	Phase 3	556	FFS
NCT02611960	NPC (Recurrent/Metastatic)	Pembrolizumab (Anti-PD-1)	Capecitabine + Gemcitabine + Docetaxel (Chemotherapy)	Phase 3	233	OS

*BSC* best supportive car, *DFS* disease-free survival, *EFS* event-free survival, *ORR* objective response rate, *OS* overall survival, *PAEs* percentage of participants with adverse events, *PFS* progression-free survival, *RFS* recurrence-free survival, *TEAEs* incidence of treatment-emergent adverse events, *TIR* time in response, *TTP* time to progression, *SAER* serious adverse event incidence rates, *SBRT* stereotactic body radiotherapy, *3D-CRT* three-dimensional conformal radiation therapy, *IMRT* intensity-modulated radiation therapy, *CCRT* concurrent chemoradiotherapy

## Data Availability

All data generated or analyzed during this study are included in this published article.

## Abbreviations

3D-CRT: Three-dimensional conformal radiation therapy
ADC: Antibody-drug conjugate
AI: Artificial intelligence
BrM: Brain metastasis
CAF: Cancer-associated fibroblast
CC: Cancer cell
CRS: Cytokine release syndrome
CSC: Cancer stem cell
DC: Dendritic cell
EBV: Epstein–Barr virus
ECM: Extracellular matrix
EMT: Epithelial–mesenchymal transformation
ESC: Esophageal cancer
GDNF: Glial cell line-derived neurotrophic factor
GFRα3: GDNF family receptor alpha 3
HBV: Hepatitis B virus
HCC: Hepatocellular carcinoma
HPD: Hyperprogressive disease
ICI: Immune checkpoint inhibitor
IDO: Indoleamine 2,3-dioxygenase
IFN-α: Interferon-α
irAEs: Immune-related adverse events
Kyn: Kynurenine
Mac: Macrophage
MANA: Mutation-associated neoantigen
MDSC: Myeloid-derived suppressor cell
MSI: Microsatellite instability
NGS: Next-generation sequencing
NK: Nature killer cells
NLR: Neutrophil-to-lymphocyte ratio
NPC: Nasopharyngeal carcinoma
NSCLC: Non-small cell lung cancer
OS: Overall survival
PD-1: Programmed cell death protein 1
PD-L1: Programmed death ligand 1
PFS: Progression-free survival
RCC: Renal cell carcinoma
RT: Radiation therapy
SBRT: Stereotactic body radiotherapy
scRNA-Seq: Single-cell RNA sequencing
sPD-L1: Soluble PD-L1
ST: Spatial transcriptomics
STIE: Systemic tumor immune environment
TAM: Tumor-associated macrophage
Teff: Effector T cells
Tex: Exhausted CD8^+^ T cells
Texp: Precursor exhausted CD8^+^ T cells
TGF-β1: Transforming growth factor-beta1
TIME: Tumor immune microenvironment
TLS: Tertiary lymphoid structure
TMB: Tumor mutation burden
TME: Tumor microenvironment
Treg: Regulatory T cell
Trp: Tryptophan
VEGF: Vascular endothelial growth factor
α-SMA: α-Smooth muscle actin
